# Associations of body shape phenotypes with sex steroids and their binding proteins in the UK Biobank cohort

**DOI:** 10.1038/s41598-022-14439-9

**Published:** 2022-06-24

**Authors:** Sofia Christakoudi, Elio Riboli, Evangelos Evangelou, Konstantinos K. Tsilidis

**Affiliations:** 1 Department of Epidemiology and Biostatistics, School of Public Health, Imperial College London, St Mary’s Campus, Norfolk Place, London, W2 1PG UK; 2 Department of Inflammation Biology, School of Immunology and Microbial Sciences, King’s College London, London, UK; 3 Department of Hygiene and Epidemiology, University of Ioannina School of Medicine, Ioannina, Greece

**Keywords:** Steroid hormones, Obesity, Epidemiology

## Abstract

Associations of sex steroids and their binding proteins with body shape are unclear, because waist and hip circumference are correlated strongly with body size. We defined body shape using “a body shape index” (ABSI) and hip index (HI), which are independent of weight and height by design, and examined associations in multivariable generalised linear models for the UK Biobank cohort (179,902 men, 207,444 women). Total testosterone was associated inversely with ABSI, especially in men. Free testosterone was lowest for large-ABSI-large-HI (“wide”) and highest for small-ABSI-small-HI (“slim”) in men, but lowest for small-ABSI-large-HI (“pear”) and highest for large-ABSI-small-HI (“apple”) in women. Oestradiol was associated inversely with ABSI in obese pre-menopausal women but positively with HI in obese men and post-menopausal women not using hormone replacement therapy. Sex-hormone binding globulin (SHBG) was associated inversely with ABSI but positively with HI and was lowest for “apple” and highest for “pear” phenotype in both sexes. Albumin was associated inversely with HI in women, but matched the pattern of free testosterone in obese men (lowest for “wide”, highest for “slim” phenotype). In conclusion, sex steroids and their binding proteins are associated with body shape, including hip as well as waist size, independent of body size.

## Introduction

Body shape shows distinct sexual dimorphisms, suggesting a key regulatory role of sex steroids^1^. Body shape also influences the consequences of obesity, with a higher risk of metabolic and cardiovascular complications for large waist size but lower risk for large hip size^2^. Body shape is further associated with a range of cancers, independent of associations with body size^3^. It is, therefore, of interest to examine associations between body shape and circulating sex steroids.

Associations of circulating sex steroids with body shape have so far been examined using the traditional anthropometric indices waist circumference (WC), hip circumference (HC), and the waist-to-hip ratio (WHR)^4–8^. Inverse associations of WHR and body mass index (BMI) with total oestradiol but positive associations with free oestradiol in pre-menopausal women, as well as positive associations with total and free testosterone and inverse associations with sex hormone binding globulin (SHBG) in all women have been reported for the UK Biobank cohort^6^. In UK Biobank men, waist circumference, WHR and BMI were associated inversely with SHBG, total and free testosterone^8^. It remains unclear, however, what is the contribution of hip size and whether there is heterogeneity of the associations with body shape according to BMI.

In addition, a major problem when examining associations with waist and hip circumference and adjusting them for BMI is their strong correlation with BMI, which may lead to biased estimates^3^. To overcome this limitation, waist and hip circumference have been scaled to weight and height in the allometric indices “a body shape index” (ABSI) and hip index (HI), which compare waist and hip circumference, correspondingly, for a given individual with the expected average for individuals with the same weight and height^9,10^. Using data from UK Biobank, we have previously described the associations of body shape phenotypes based on ABSI and HI with allometric body-composition indices, which similarly to ABSI and HI normalise body composition measurements for weight and height^11^. An analogous approach, normalising body composition measurements for height, BMI, and waist circumference has been used for the 1999–2006 United States National Health and Nutrition Examination Survey (NHANES)^12^.

In the current study, we have examined the association of body shape phenotypes based on allometric indices with serum levels of oestradiol and testosterone and their binding proteins (SHBG and albumin) and have explored heterogeneity by BMI, separately in men and women from the UK Biobank cohort, defining in men subgroups according to age and in women subgroups according to menopausal status and use of hormone replacement therapy (HRT).

## Methods

### Study population

The UK Biobank cohort includes half a million participants recruited between 2006 and 2010 from the general population of the United Kingdom (UK), at age 40–70 years^13^. Similar to our previous studies^3,11^, we have included in the current study only participants with self-reported white ancestry, because the number of participants with other ethnicities was relatively small. In total, we excluded 115,113 participants with missing measurements for testosterone and oestradiol, extreme or missing anthropometric measurements, mismatched genetic and self-reported sex, prevalent cancer at enrolment (defined as previously described^3^), incident cancer or death within the first two years after enrolment (to minimise the influence of cancer cachexia), women pregnant at enrolment, participants receiving glucocorticoids or drugs influencing the production or action of sex steroids (listed in Supplementary Table S1), men receiving testosterone or 5α-reductase inhibitors and women with missing information for use of oral contraceptives or HRT. We examined separately men and women overall and defined groups in men by age (< 55 years and ≥ 55 years) and in women by self-reported menopausal status (excluding undetermined) and HRT use (see the definitions of variables in Supplementary Methods), with an additional restriction by age at enrolment as follows: Pre-MP—pre-menopausal women < 55 years of age, who had never used HRT and were not using oral contraceptives or cholesterol lowering drugs at enrolment (the latter may affect de novo sex steroid synthesis, as this requires cholesterol); Post-MP—post-menopausal women ≥ 50 years of age, which were not receiving oral contraceptives at enrolment and had never used HRT (Post-MP Never-HRT), or had used HRT in the past (Post-MP Past-HRT), or were current HRT users at enrolment (Post-MP Current-HRT). Supplementary Fig. S1 shows a flow diagram of the exclusions, with the numbers excluded at each step and the final number of participants in each examined group.

### Anthropometric indices

Anthropometric measurements were obtained by UK Biobank, at the natural indent or the umbilicus for waist circumference and at the widest point for hip circumference^13,14^. We calculated ABSI for both sexes with coefficients from NHANES 1999–2004^9^, but using waist circumference in mm and not in m as in the original formula, to derive ABSI values in the order of magnitude of waist circumference. We calculated HI for women with coefficients from NHANES III^10^, but HI for men with simple-fraction coefficients based on UK Biobank data^11^, to correct the underlying inverse correlation between HI calculated with the original coefficients and BMI observed in UK Biobank men^3^.

$$\begin{aligned} {\text{ABSI}} &= {\text{WC }} ({{\text{mm}}})\times {\text{Weight }} ({{\text{kg}}} )^{{ - {2}/{3}}} \times {\text{Height }} ({\text{m}})^{{{5}/{6}}} \\ {\text{HI}}_{{{\text{women}}}} &= {\text{ HC }} ( {{\text{cm}}} )\times {\text{Weight }} ({{\text{kg}}})^{{ - 0.482}} \times {\text{Height }} ( {{\text{cm}}} )^{{0.310}} \\ {\text{HI}}_{{{\text{men}}}} &= {\text{ HC }} ( {{\text{cm}}})\times {\text{Weight }} ( {{\text{kg}}})^{{ - {2}/{5}}} \times {\text{Height }} ({{\text{cm}}})^{{{1}/{5}}} \\ {\text{BMI }} &= {\text{ Weight }} ( {{\text{kg}}} ) \times {\text{Height }}( {\text{m}})^{{ - {2}}} \end{aligned}$$

To standardise anthropometric indices on a continuous scale, we calculated sex-specific z-scores (value minus mean, divided by standard deviation, SD). To define categories of BMI, we used World Health Organisation cut-offs: normal weight (BMI ≥ 18.5 to < 25 kg/m^2^), overweight (BMI ≥ 25 to < 30 kg/m^2^), and obese (BMI ≥ 30 to < 45 kg/m^2^). To define categories of body shape, we used numerical cut-offs for ABSI (≥ 80 for men, ≥ 73 for women) and HI (≥ 49 for men, ≥ 64 for women), which in our previous study on body composition were close to the sex-specific UK Biobank medians^11^. This dichotomisation of ABSI and HI on an absolute scale, similarly to the definition of WHO categories of BMI, permits comparisons between different populations, while a z-score-based classification would be dependent on the mean and SD of each population. Also as previously^11^, we used the dichotomised ABSI and HI to define an ABSI-by-HI cross-classification with four body shape phenotypes named “wide”—large-ABSI-large-HI, “apple”—large-ABSI-small-HI, “slim”—small-ABSI-small-HI, and “pear”—small-ABSI-large-HI (mnemonic “WASP”). On the same principle, a two-by-two cross-classification has previously been defined for body composition by dichotomising the percentages of trunk and leg fat mass at their means (z-score > 0)^15^. In this, however, the “apple” group comprised all individuals with large trunk fat mass, irrespective of leg fat mass, and the “pear” group comprised all individuals with large leg fat mass, irrespective of trunk fat mass, thus including individuals with large trunk and large leg fat mass in both the “apple” and the “pear” groups. In our definition, we have adopted an opposition of the male and female body shape patterns^1^, defining “apple” and “pear” as non-overlapping groups, which include individuals with discordant waist and hip size, and separating these from the “slim” and “wide” groups, which include individuals with concordant waist and hip size.

### Biomarker measurements

Serum levels of oestradiol, testosterone and SHBG were measured with chemiluminescent immunoassays (competitive binding for sex steroids, two-step sandwich for SHBG) on Beckman Coulter DXI 800 analyser. Albumin levels were measured with a colorimetric assay on Beckman Coulter AU5800 analyser. All measurements were provided by UK Biobank. Levels were below the limit of detection for 15.2% of testosterone measurements in women and for the majority of oestradiol measurements in men and post-menopausal women (Table 1). To accommodate undetected values, we used total oestradiol for men and post-menopausal women as a binary variable, dichotomised at the lowest detected level (175 pmol/L), and imputed total oestradiol for Pre-MP women (22.6% of measurements undetected) and total testosterone for all women after log-transformation (Supplementary Methods). We used quantile regression imputation of truncated left-censored data, which estimates the parameters of the distribution of the available data and uses these to impute the left tail below the limit of detection (QRILC, imputeLCMD v2.0 package in R). Biomarker results above the upper limit of detection were few and were set to the upper limit value. We calculated free testosterone for all and free oestradiol for pre-menopausal women using the affinity constants and law-of-mass-action equations reported by Sodergard et al*.*^16^ (Supplementary Methods). We log-transformed all biomarker measurements and standardised them to sex-specific z-scores.

**Table 1:** Anthropometric characteristics of study participants and biomarker levels.

	Men			Women				
	Overall	< 55 years	≥ 55 years	Overall	Pre-MP	Post-MP Never-HRT	Post-MP Past-HRT	Post-MP Current-HRT
Cohort: n (% per sex)	179,902	69,378 (38.6)	110,524 (61.4)	207,444	40,956 (19.7)	63,134 (30.4)	54,591 (26.3)	10,722 (5.2)
Age (years)^a^	57 (8.1)	48.2 (4.2)	62.6 (4.1)	56.8 (8.0)	46.4 (3.6)	60.2 (5.2)	62.4 (4.4)	59.7 (5.1)
Hand grip strength^a^	42.1 (8.9)	44.8 (9.0)	40.5 (8.3)	25.3 (6.3)	28.5 (6.1)	24.5 (5.9)	23.5 (5.9)	24.7 (6.1)
BMI (kg/m^2^)^a^	27.8 (4.0)	27.7 (4.1)	27.8 (4.0)	26.9 (4.8)	26.1 (4.8)	27.0 (4.8)	27.3 (4.6)	26.3 (4.3)
**BMI categories**								
NW: n (%)	45,001 (25.0)	18,614 (26.8)	26,387 (23.9)	83,710 (40.4)	20,235 (49.4)	24,996 (39.6)	19,247 (35.3)	4764 (44.4)
OW: n (%)	89,761 (49.9)	33,802 (48.7)	55,959 (50.6)	77,227 (37.2)	13,224 (32.3)	23,820 (37.7)	22,204 (40.7)	4092 (38.2)
OB: n (%)	45,140 (25.1)	16,962 (24.4)	28,178 (25.5)	46,507 (22.4)	7497 (18.3)	14,318 (22.7)	13,140 (24.1)	1866 (17.4)
**Body shape indices**								
Waist circumference^a^	96.8 (10.9)	95.5 (10.9)	97.6 (10.9)	84.2 (11.9)	81.5 (11.5)	84.7 (12.0)	85.6 (11.6)	82.8 (11.0)
Hip circumference^a^	103.4 (7.2)	103.4 (7.2)	103.4 (7.2)	103.1 (9.7)	102.1 (9.5)	103.1 (9.7)	103.5 (9.5)	101.8 (8.8)
ABSI^a^	79.7 (4.1)	78.6 (3.9)	80.4 (4.0)	73.8 (4.9)	72.4 (4.7)	74.2 (5.0)	74.5 (4.9)	73.7 (4.9)
HI^a^	49.1 (1.7)	49.0 (1.6)	49.2 (1.7)	64.2 (2.5)	64.1 (2.4)	64.3 (2.5)	64.3 (2.5)	64.2 (2.4)
**ABSI-by-HI**								
Pear: n (%)	43,199 (24.0)	20,187 (29.1)	23,012 (20.8)	50,114 (24.2)	12,230 (29.9)	14,233 (22.5)	11,429 (20.9)	2607 (24.3)
Slim: n (%)	51,544 (28.7)	24,511 (35.3)	27,033 (24.5)	44,661 (21.5)	11,330 (27.7)	12,439 (19.7)	9894 (18.1)	2302 (21.5)
Wide: n (%)	51,978 (28.9)	14,714 (21.2)	37,264 (33.7)	63,358 (30.5)	9441 (23.1)	20,850 (33.0)	18,981 (34.8)	3209 (29.9)
Apple: n (%)	33,181 (18.4)	9966 (14.4)	23,215 (21.0)	49,311 (23.8)	7955 (19.4)	15,612 (24.7)	14,287 (26.2)	2604 (24.3)
**Biomarkers**								
SHBG (nmol/L)^b^	36.4 (16.1–82.3)	32.0 (14.1–72.8)	39.5 (18.3–85.3)	55.8 (21.1–148)	61.7 (25.3–151)	53.0 (21.4–132)	52.0 (21.4–126)	68.3 (19.5–240)
Albumin (g/L)^b^	45.5 (40.7–50.9)	46.2 (41.6–51.4)	45.1 (40.4–50.3)	45 (40.2–50.3)	44.9 (40.2–50.2)	45.1 (40.4–50.4)	45.0 (40.3–50.2)	44.3 (39.5–49.8)
T (nmol/L)^b^	11.5 (6.2–21.2)	11.8 (6.5–21.4)	11.3 (6.1–21.0)	0.7 (0.1–4.4)	1.0 (0.3–3.9)	0.8 (0.1–4.2)	0.6 (0.1–4.2)	0.6 (0.1–4.1)
Free T (pmol/L)^b^	240 (141–409)	258 (154–430)	230 (136–389)	12.0 (1.9–76.8)	15.1 (3.4–66.7)	12.6 (2.1–75.4)	10.7 (1.5–75.0)	7.9 (0.9–68.4)
E2 (detected): n (%)	15,048 (8.4)	6003 (8.7)	9045 (8.2)	48,835 (23.5)	29,646 (72.4) ^c^	2023 (3.2)	980 (1.8)	3628 (33.8)

*ABSI* a body shape index (cut-offs ≥ 80 for men, ≥ 73 for women), *Apple* large-ABSI-small-HI, *BMI* body mass index, *E2* oestradiol, *HI* hip index (cut-offs ≥ 49 for men, ≥ 64 for women), *HRT* hormone replacement therapy, *NW* normal weight (BMI ≥ 18.5 to < 25 kg/m^2^), *OB* obese (BMI ≥ 30 to < 45 kg/m^2^), *OW* overweight (BMI ≥ 25 to < 30 kg/m^2^), *Pear* small-ABSI-large-HI, *Post-MP* post-menopausal, *Pre-MP* pre-menopausal, *SHBG* sex hormone binding globulin, *Slim* small-ABSI-small-HI, *T* testosterone, *Wide* large-ABSI-large-HI, *n* (*%*) number (percentage from total per column); ^a^mean (standard deviation); ^b^geometric mean (95% reference range); ^c^for Pre-MP women, E2 (pmol/L) was 318 (50–2007) and free E2 (pmol/L) was 6.5 (1.1–39.6). Participant groups are defined in Supplementary Fig. S1.

### Statistical analysis

We used multivariable linear regression models to calculate SD differences (95% confidence interval), except for dichotomised oestradiol, for which we used multivariable logistic regression models and estimated odds ratios of oestradiol detection (95% confidence interval). We first examined anthropometric indices as continuous variables (sex-specific z-scores, per one SD increment) in an additive model including ABSI, HI, BMI, and covariates. We then examined anthropometric indices as categorical variables, in an additive model including body shape phenotypes, defined with the ABSI-by-HI cross-classification, BMI categories and covariates. Body shape phenotypes were ordered according to increasing visceral adipose tissue (VAT) quantity: “pear” (reference), “slim”, “wide”, “apple”^11^. Finally, we examined heterogeneity of the associations with body shape according to body size with a model including a BMI-by-ABSI-by-HI cross-classification (“pear” normal weight reference) and covariates and with models including dichotomised ABSI and HI (large vs small, using the same cut-offs as for body shape phenotypes) and covariates within subgroups according to BMI categories. Similar to our previous studies^3,11^, covariates comprised height, age at enrolment, weight change within the last year preceding enrolment, smoking status, alcohol consumption, physical activity, Townsend deprivation index, region of the assessment centre, and additionally, time of blood collection, fasting time, use of cholesterol lowering drugs (except Pre-MP), and in women also age at the last live birth, oral contraceptives use with time since stopped, bilateral oophorectomy (except Pre-MP), and additionally menopausal status and HRT use and duration (women overall), time of the menstrual period (Pre-MP), time since stopped and duration of HRT use (Past-HRT), or duration of use and type of HRT (Current-HRT) (covariates are defined in Supplementary Methods). To examine associations between sex steroids and their binding proteins, we calculated partial Pearson correlation coefficients with adjustment for ABSI, HI, BMI, and height (continuous, z-scores), and covariates as above (except for region and using Townsend deprivation index, time of blood collection, and fasting time on a continuous scale). We used the median category per sex in place of missing values for covariates, which were limited (Supplementary Table S2).

We tested the contribution of body shape phenotypes overall with a likelihood ratio test, comparing a model including BMI categories and covariates with a model additionally including an ABSI-by-HI cross-classification. Individual terms were evaluated with the corresponding Wald test. We tested heterogeneity of the associations with body shape phenotypes by BMI with a likelihood ratio test, comparing an additive model including the ABSI-by-HI cross-classification, BMI categories, and covariates with an interaction model including the BMI-by-ABSI-by-HI cross-classification and covariates. We tested heterogeneity of the associations of ABSI and HI by BMI with a likelihood ratio test, comparing an additive model including dichotomised ABHI and HI, BMI categories, and covariates with a model including an interaction term between ABSI and BMI (or between HI and BMI) and covariates. Tests of statistical significance were two-sided, with p < 0.05 considered a weaker evidence and p < 0.0001 a stronger evidence.

We used R version 4.1.0 for all analyses^17^.

### Ethical approval and consent to participate

This research was conducted according to the principles expressed in the Declaration of Helsinki. The UK Biobank cohort has been approved by the North West Multicenter Research Ethics Committee, UK (Ref: 16/NW/0274). Written informed consent has been obtained from all study participants. The current study was approved by the UK Biobank access management board. Participants who had withdrawn consent by the time of the analysis were excluded from dataset.

## Results

### Cohort characteristics

In total, 179,902 men and 207,444 women were included in the study. Younger men and pre-menopausal women were less likely to have stable weight but had higher hand grip strength, lower BMI, smaller ABSI, and a smaller proportion of “apple” and “wide” phenotypes compared, correspondingly, to older men and post-menopausal women (Table 1, Supplementary Table S2). Younger men and pre-menopausal women were also less likely to consume alcohol daily, to use cholesterol lowering drugs, or to smoke regularly in the past but were more likely to be current smokers, physically active, with higher Townsend deprivation index, and pre-menopausal women were also less likely to have children or to have them at a younger age and more likely to use oral contraceptives (Supplementary Table S2). Post-menopausal women currently using HRT had lower BMI, comparable to pre-menopausal women, but their hand grip strength and body shape patterns were comparable to post-menopausal women never or past HRT users (Table 1). Post-menopausal women with past or current HRT use were more likely to be younger at menopause or to have bilateral oophorectomy, and to be current or former smokers, to consume alcohol daily, or to have used oral contraceptives in the past, compared to never HRT users (Supplementary Table S2). Post-menopausal women with past HRT use had the highest BMI and the largest proportion of “apple” and “wide” phenotypes but the lowest hand grip strength among women, and were most likely to have their children at an earlier age or to use cholesterol lowering drugs (Table 1, Supplementary Table S2).

**Figure 1:**
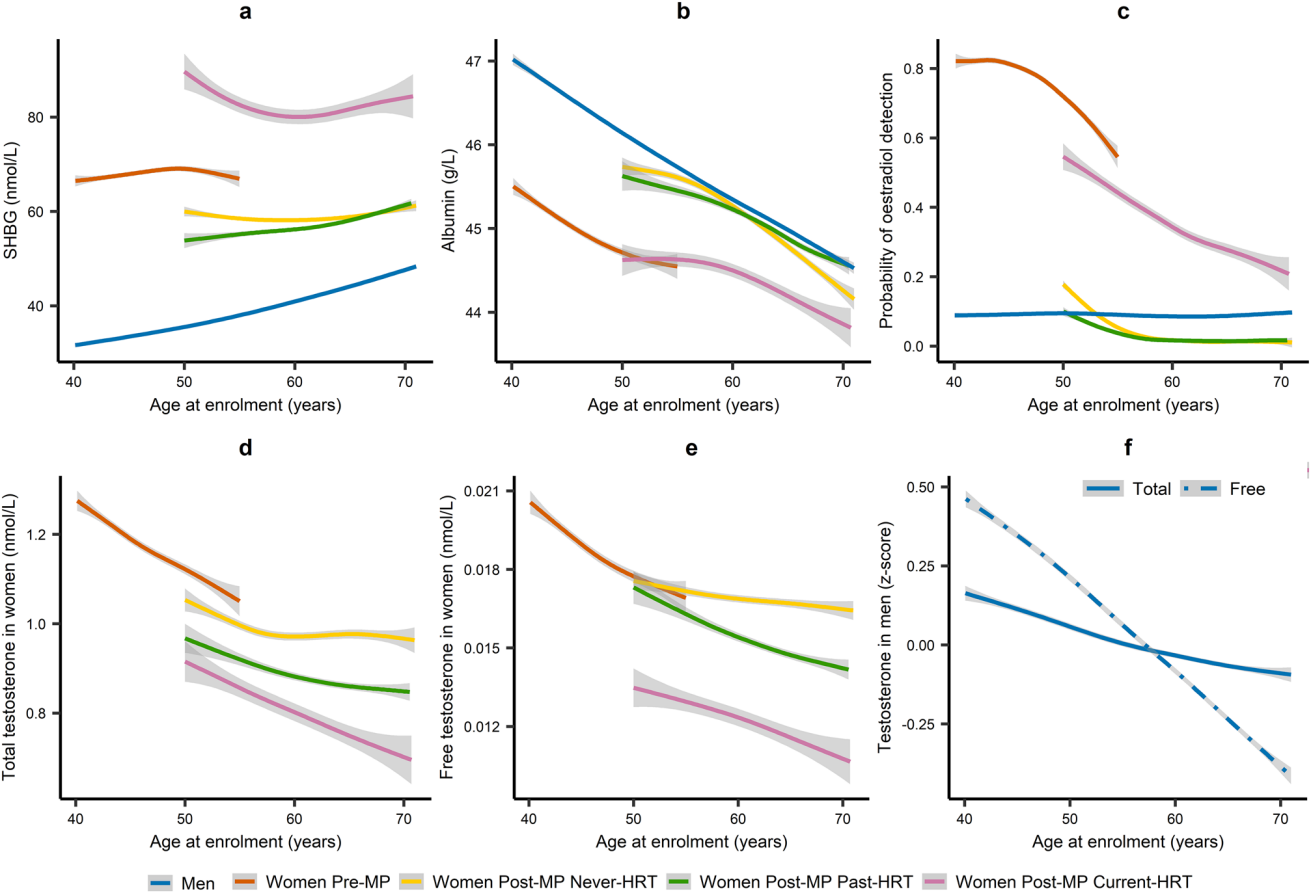
Sex steroids and their binding proteins according to age at enrolment. *HRT* hormone replacement therapy, *Post-MP* post-menopausal, *Pre-MP* pre-menopausal, *SHBG* sex hormone binding globulin, *z-score* value minus mean divided by standard deviation, sex specific. Plots represent means of unadjusted levels (95% confidence interval), determined with the generalised additive models smoothing function from package mgcv, with restricted maximum likelihood (REML) estimation, applied as default for large datasets in ggplot2 v.3.3.5 in R v4.1.0. Participant groups are defined in Supplementary Fig. S1. Estimates for associations with age obtained from fully adjusted multivariable models are shown in Supplementary Table S3.

Older men had higher SHBG compared to younger men, but men had lower levels compared to women and the highest SHBG levels were in women with current HRT use (Fig. 1a). In both sexes, albumin was lower for older age, but the lowest levels were in pre-menopausal women and in post-menopausal women with current HRT use (Fig. 1b). In women, total and free testosterone, as well as oestradiol, were lower for older age, even with HRT use (Fig. 1c–e). Post-menopausal women with never or past HRT use had lower total oestradiol than men (Fig. 1c). In men, total oestradiol was similar for all ages (Fig. 1c), while free testosterone more than total testosterone was lower for older age (Fig. 1f). Associations with age for the unadjusted levels (Fig. 1) were consistent with fully adjusted models (Supplementary Table S3).

### Associations of body size with sex steroids and their binding proteins

**Figure 2:**
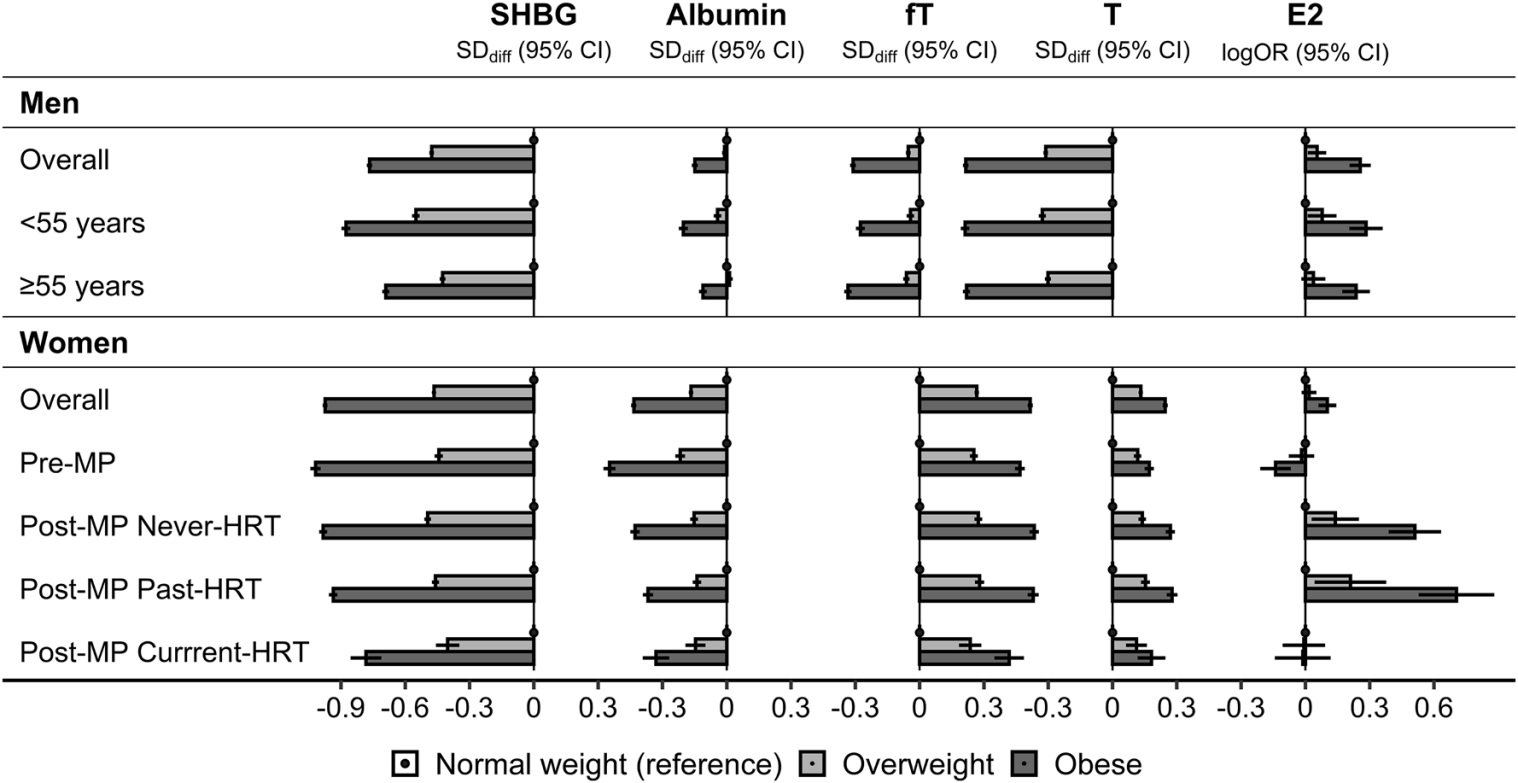
Associations of body size with sex steroids and their binding proteins. *BMI* body mass index, *E2* oestradiol, *fT* free testosterone, *HRT* hormone replacement therapy, *Normal weight* BMI ≥ 18.5 to < 25.0 kg/m^2^, *Obese* BMI ≥ 30 to < 45 kg/m^2^, *Overweight* BMI ≥ 25 to < 30 kg/m^2^, *Post-MP* post-menopausal, *Pre-MP* pre-menopausal, *SHBG* sex hormone binding globulin, *T* total testosterone, *SD*_*diff*_ (*95% CI*) estimates for standard deviation differences (95% confidence interval) were obtained from multivariable linear regression models including SHBG, albumin, T, or fT on a continuous standard deviation scale (sex-specific z-scores) as an outcome variable and as independent variables BMI categories and covariates, *logOR* (*95% CI*) estimates for odds ratios of oestradiol detection (95% confidence interval) were obtained from multivariable logistic regression models (logOR corresponds to the linear predictor or the model). Covariates included an ABSI-by-HI cross-classification, height, age at enrolment, weight change within the last year preceding enrolment, smoking status, alcohol consumption, physical activity, Townsend deprivation index, region of the assessment centre, time of blood collection, fasting time, use of cholesterol lowering drugs (except Pre-MP), and in women also age at the last live birth, oral contraceptives use with time since stopped, bilateral oophorectomy (except Pre-MP) and, additionally, menopausal status and HRT use and duration (women overall), time of the menstrual period (Pre-MP), time since stopped and duration of HRT use (Past-HRT), or duration of use and type of HRT (Current-HRT). Participant groups are defined in Supplementary Fig. S1. Covariates are defined in Supplementary Methods. Numerical values are shown in Supplementary Table S5.

SHBG was strongly inversely associated with BMI in men and women, even in women with current HRT use (Supplementary Table S4 for BMI on a continuous scale, Fig. 2 for BMI categories). Albumin was also associated inversely with BMI, but the associations were stronger in women compared to men, in pre-menopausal compared to post-menopausal women, and in younger compared to older men. Total and free testosterone were associated inversely with BMI in men and positively in women, with a stronger association for total testosterone in men but for free testosterone in women. Total oestradiol was associated positively with BMI in men and more strongly in post-menopausal women with never or past HRT use, with no evidence for association in post-menopausal women with current HRT use. In pre-menopausal women, oestradiol was associated weakly with BMI, inversely for total oestradiol (Fig. 2) but positively for free oestradiol (Supplementary Table S4, Supplementary Table S5 for BMI categories).

### Associations of body shape with sex steroids and their binding proteins

**Figure 3:**
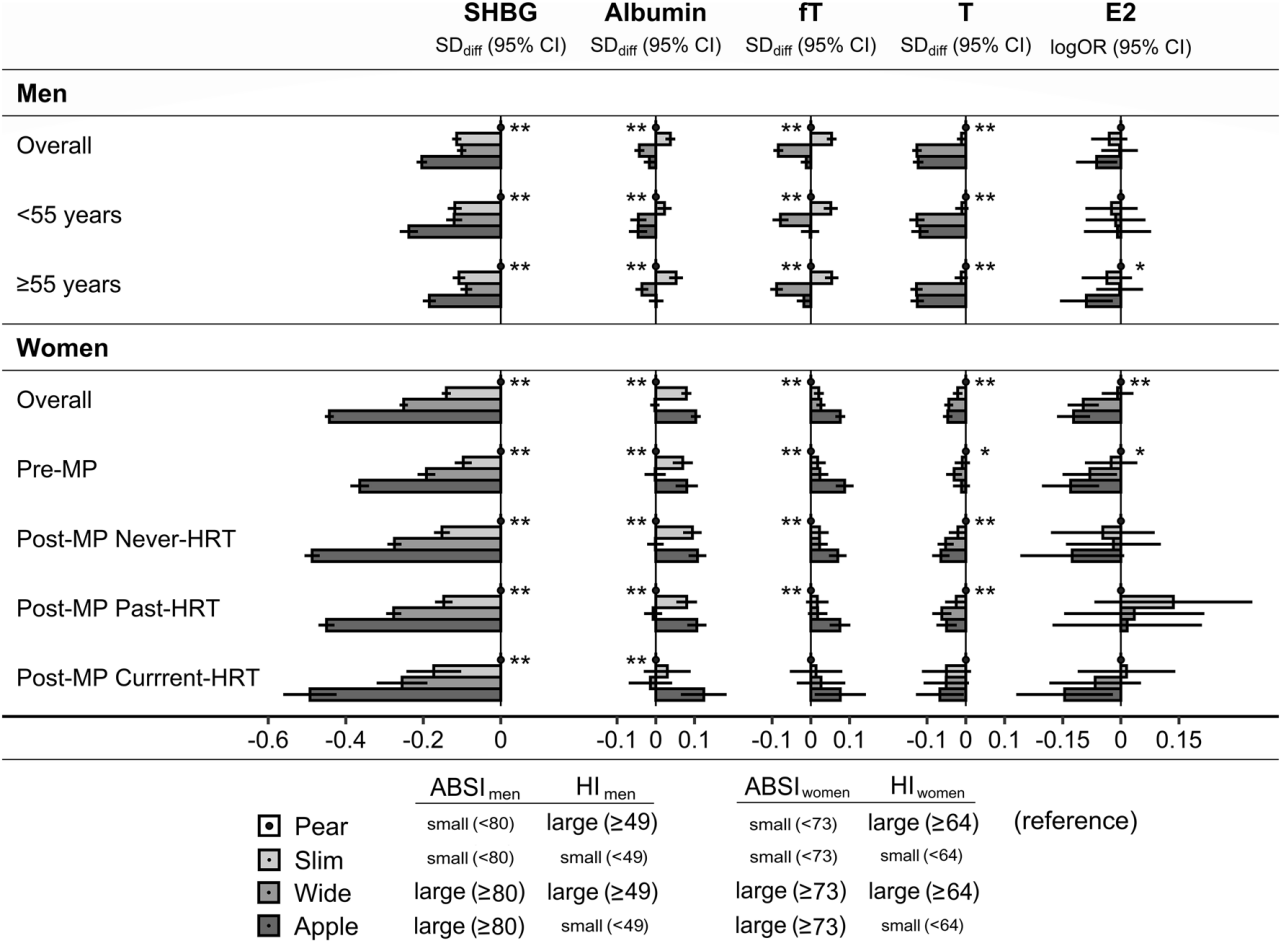
Associations of body shape phenotypes with sex steroids and their binding proteins. *ABSI* a body shape index (cut-offs ≥ 80 for men, ≥ 73 for women), *Apple* large-ABSI-small-HI, *BMI* body mass index, *E2* oestradiol, *fT* free testosterone, *HI* hip index (cut-offs ≥ 49 for men, ≥ 64 for women), *HRT* hormone replacement therapy, *Pear* small-ABSI-large-HI, *Post-MP* post-menopausal, *Pre-MP* pre-menopausal, *Slim* small-ABSI-small-HI, *SHBG* sex hormone binding globulin, *T* total testosterone, *Wide* large-ABSI-large-HI. *SD*_*diff*_ (*95% CI*) estimates for standard deviation differences (95% confidence interval) were obtained from multivariable linear regression models including SHBG, albumin, T, or fT on a continuous standard deviation scale (sex-specific z-scores) as an outcome variable and as independent variable an ABSI-by-HI cross-classification; *logOR* (*95% CI*) estimates for the odds ratios of oestradiol detection (95% confidence interval) were obtained from multivariable logistic regression models (logOR corresponds to the linear predictor of the model). All models were adjusted for BMI categories, height, age at enrolment, weight change within the last year preceding enrolment, smoking status, alcohol consumption, physical activity, Townsend deprivation index, region of the assessment centre, time of blood collection, fasting time, use of cholesterol lowering drugs (except Pre-MP), and in women also age at the last live birth, oral contraceptives use with time since stopped, bilateral oophorectomy (except Pre-MP) and, additionally, menopausal status and HRT use and duration (women overall), time of the menstrual period (Pre-MP), time since stopped and duration of HRT use (Past-HRT), or duration of use and type of HRT (Current-HRT). Participant groups are defined in Supplementary Fig. S1. Covariates are defined in Supplementary Methods. Numerical values are shown in Supplementary Table S6. *p < 0.05 from a likelihood ratio test comparing a model including only a categorical variable for BMI and covariates with a model additionally including an ABSI-by-HI cross-classification variable (evaluates body shape overall); **p < 0.0001.

Independent of BMI, SHBG was associated inversely with waist size evaluated with ABSI and positively with hip size evaluated with HI in men and women, including in women with current HRT use (Supplementary Table S4 for ABSI and HI on a continuous scale). Correspondingly, SHBG levels were lowest for “apple” and highest for “pear” phenotype in both sexes, with intermediate levels for “wide” and “slim” phenotypes, which were similar in men but lower for “wide” compared to “slim” phenotype in women (Fig. 3 for body shape phenotypes). Albumin showed weak inverse associations with HI in women (highest for “slim” and “apple” phenotypes), with ABSI in younger men (lowest for “wide” and “apple” phenotypes), and with ABSI and HI in older men (lowest for “wide” and highest for “slim” phenotype). Total testosterone was associated inversely with ABSI (lowest for “wide” and “apple” phenotypes), more prominently in men than in post-menopausal women, but not in pre-menopausal women. The association patterns of free testosterone with body shape differed between women and men. In women, including pre-menopausal, free testosterone was associated weakly positively with ABSI and inversely with HI, with the highest levels for “apple” phenotype. In men, free testosterone was associated inversely with both ABSI and HI, with the lowest levels for “wide” and the highest for “slim” phenotype, and with no material difference between “pear” and “apple” phenotypes. Associations of total oestradiol with body shape overall were weak, mainly inverse with ABSI, with the lowest levels for “apple” phenotype, especially in pre-menopausal women but not in younger men or post-menopausal women with past HRT use (Fig. 3). There was little evidence for associations of free oestradiol with body shape in pre-menopausal women (Supplementary Table S6).

### Heterogeneity of the associations of body shape with sex steroids and their binding proteins according to body size

**Figure 4:**
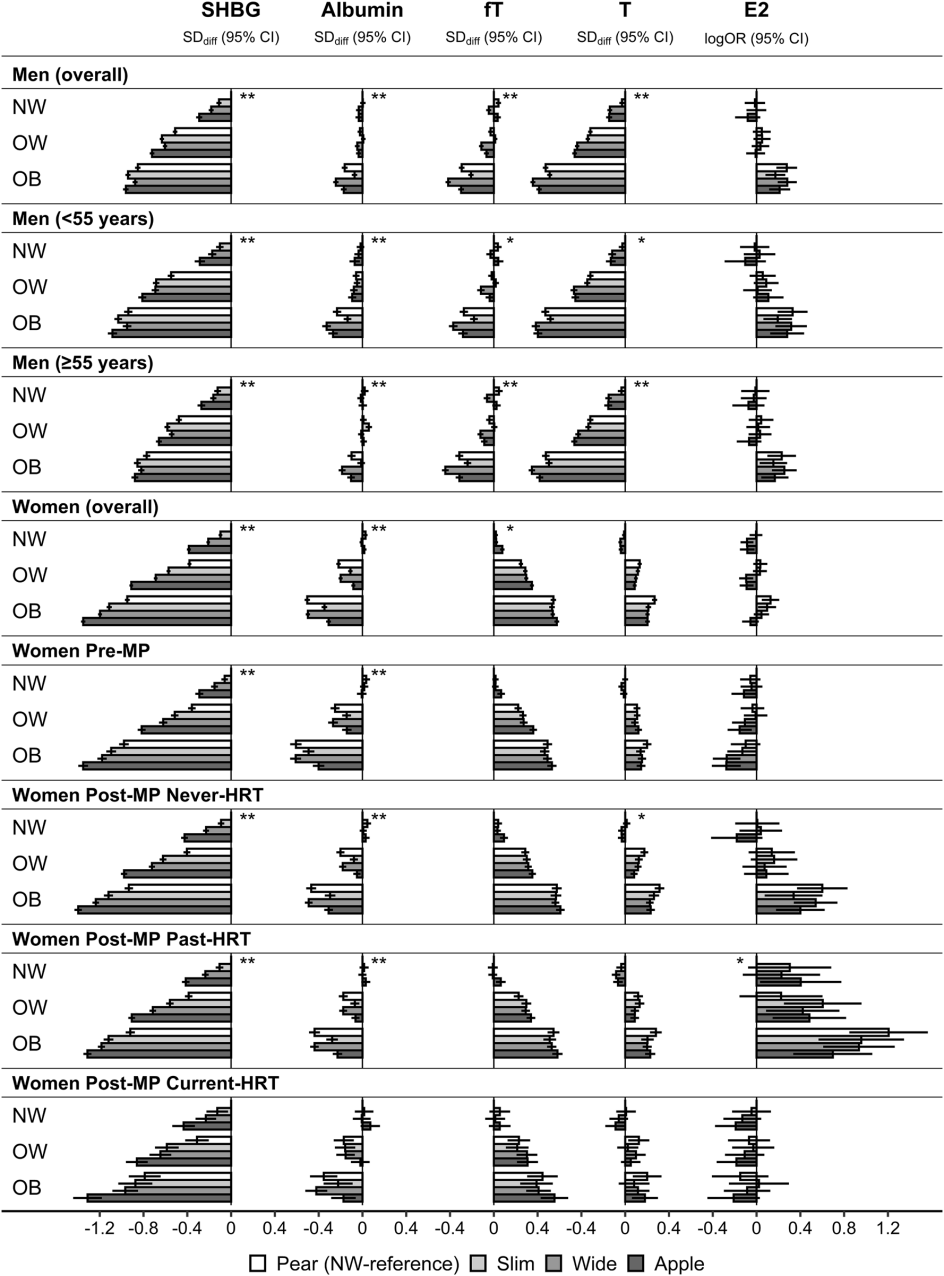
Associations of body shape phenotypes with sex steroids and their binding proteins: heterogeneity by BMI. *ABSI* a body shape index (cut-offs ≥ 80 for men, ≥ 73 for women), *Apple* large-ABSI-small-HI, *BMI* body mass index, *E2* oestradiol, *fT* free testosterone, *HI* hip index (cut-offs ≥ 49 for men, ≥ 64 for women), *HRT* hormone replacement therapy, *NW* normal weight (BMI ≥ 18.5 to BMI < 25 kg/m^2^), *OB* obese (BMI ≥ 30 to BMI < 45 kg/m^2^), *OR* odds ratio, *OW* overweight (BMI ≥ 25 to BMI < 30 kg/m^2^), *Pear* small-ABSI-large-HI, *Post-MP* post-menopausal, *Pre-MP* pre-menopausal, *Slim* small-ABSI-small-HI, *SHBG* sex hormone binding globulin, *T* total testosterone, *Wide* large-ABSI-large-HI. *SD*_*diff*_ (*95% CI*) estimates for standard deviation differences (95% confidence interval) were obtained from multivariable linear regression models including SHBG, albumin, T, or fT (continuous standard deviation scale, sex-specific z-scores) as an outcome variable and BMI-by-ABSI-by-HI cross-classification as an independent variable, *logOR* (*95% CI*) estimates for odds ratios of oestradiol detection (95% confidence interval) were obtained from multivariable logistic regression models (logOR corresponds to the linear predictor of the model). All models were adjusted for height, age at enrolment, weight change within the last year preceding enrolment, smoking status, alcohol consumption, physical activity, Townsend deprivation index, region of the assessment centre, time of blood collection, fasting time, use of cholesterol lowering drugs (except Pre-MP), and in women age at the last live birth, oral contraceptives use with time since stopped, bilateral oophorectomy (except Pre-MP) and, additionally, menopausal status and HRT use and duration (women overall), time of the menstrual period (Pre-MP), time since stopped and duration of HRT use (Past-HRT), or duration of use and type of HRT (Current-HRT). Participant groups are defined in Supplementary Fig. S1. Covariates are defined in Supplementary Methods. Numerical values are shown in Supplementary Table S6. *p < 0.05 from a likelihood ratio test comparing an additive model including ABSI-by-HI cross-classification, BMI categories and covariates with an interaction model including BMI-by-ABSI-by-HI cross-classification and covariates (evaluates heterogeneity by BMI); **p < 0.0001.

In women, SHBG was lowest for “apple” and highest for “pear” phenotype for all BMI categories (Fig. 4 for body shape phenotypes by BMI categories), but associations with ABSI and HI were strongest for overweight BMI (Supplementary Fig. S2 for dichotomised ABSI and HI by BMI categories). In men, SHBG was lowest for “apple” and highest for “pear” phenotype only for normal weight and overweight BMI, while there was mainly a positive association with HI for obese BMI (highest levels for “pear” and “wide” phenotypes, Fig. 4) and the association with ABSI was attenuated for overweight and very weak for obese BMI (Supplementary Fig. S2). Albumin was associated inversely with HI in overweight and more strongly in obese women, while in men, the inverse associations of albumin with ABSI and HI, were prominent only for obese BMI, with the lowest levels for “wide” and the highest for “slim” phenotype in both younger and older men (Fig. 4, Supplementary Fig. S2). The association patterns of free and total testosterone with body shape were hard to distinguish in women (Fig. 4). In men, the inverse association of total testosterone with ABSI was broadly retained for all BMI categories, but there was an additional weak inverse association with HI for obese BMI, especially in older men (Supplementary Fig. S2). The inverse association of free testosterone with ABSI, however, was weak for normal weight BMI, especially in younger men, while the inverse association with HI was stronger for obese BMI, especially in older men (Supplementary Fig. S2). Total oestradiol showed associations with body shape mainly for obese BMI, with an inverse association with ABSI in pre-menopausal women (lowest levels for “wide” and “apple” phenotypes), a positive association with HI in men and post-menopausal women never using HRT (highest levels for “pear” and “wide” phenotypes), and an inverse association with ABSI and positive with HI in post-menopausal women with past HRT use (lowest levels for “apple” and highest for “pear” phenotype) (Fig. 4).

### Associations between sex steroids and their binding proteins

**Figure 5:**
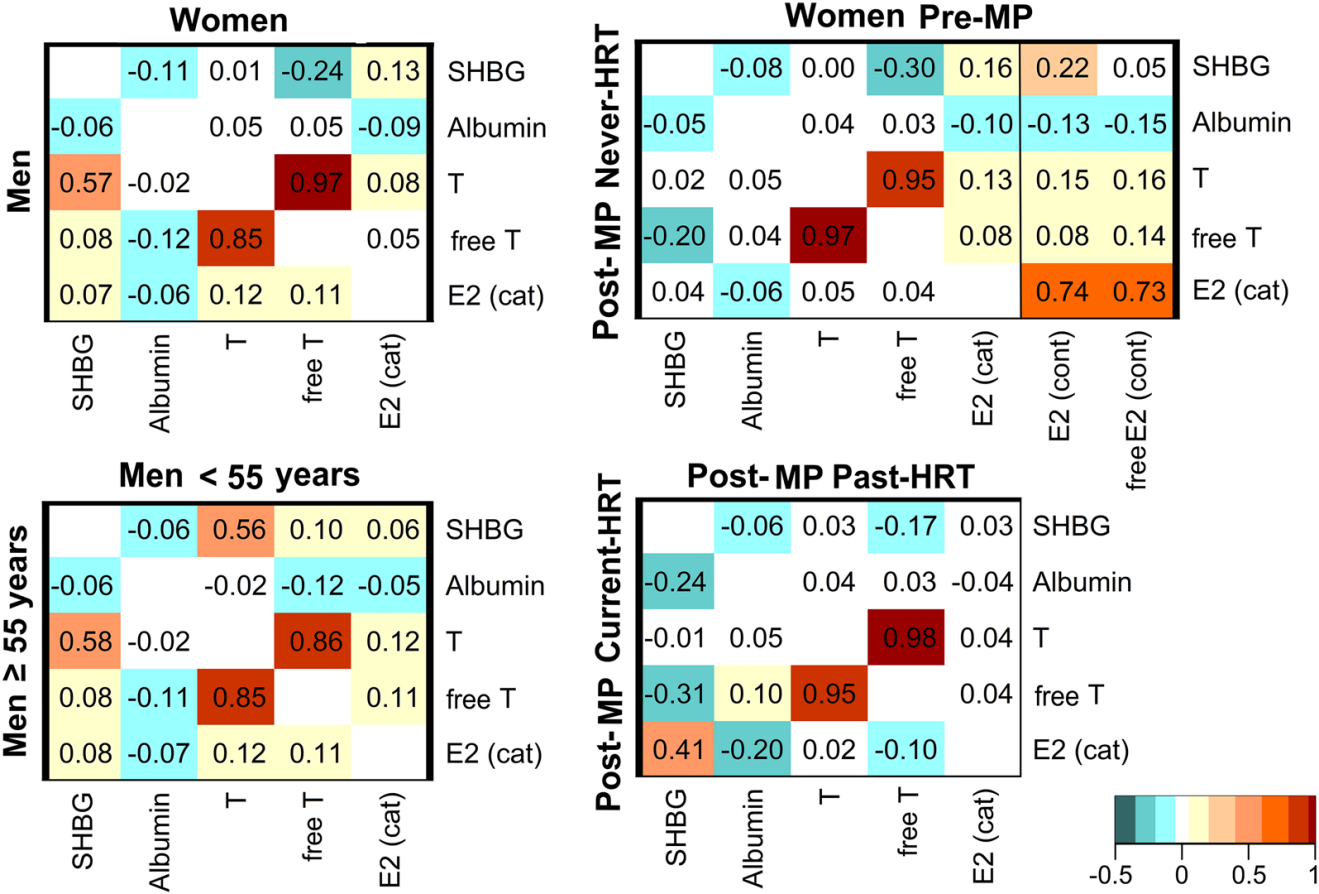
Associations between sex steroids and their binding proteins. *E2* (*cat*) oestradiol as a binary variable (undetectable/detectable), *E2* (*cont*) oestradiol as a continuous variable (with imputed values below the lower limit of detection), *free E2* free oestradiol, *free T* free testosterone, *HRT* hormone replacement therapy, *Post-MP* post-menopausal, *Pre-MP* pre-menopausal, *SHBG* sex hormone binding globulin, *T* testosterone. Pearson partial correlation coefficients were adjusted for “a body shape index” (ABSI), hip index (HI), body mass index (BMI), height (sex-specific z-scores, continuous scale), age at enrolment, weight change during the last year preceding enrolment, smoking status, alcohol consumption, physical activity, Townsend deprivation index (continuous), time of blood collection (continuous), fasting time (continuous), use of cholesterol lowering drugs (except Pre-MP), and in women age at the last live birth, oral contraceptive use, bilateral oophorectomy (except Pre-MP) and, additionally, menopausal status and HRT use (women overall), time of the menstrual period (Pre-MP), duration of use and time since stopped HRT (Past-HRT), duration of use and type of HRT (Current-HRT). Participant groups are defined in Supplementary Fig. S1. The correlation coefficient for total vs free oestradiol in Pre-MP women was 0.98. All p-values were p < 0.0001, except for SHBG vs testosterone in women (overall, p = 0.016), Pre-MP (p = 0.87) and Post-MP Current-HRT (p = 0.16), and for oestradiol vs testosterone in women Post-MP Current-HRT (p = 0.065).

Independent of body shape, body size, age, and other covariates, SHBG showed a prominent positive correlation with total testosterone in men but not in women and an inverse correlation with free testosterone in women but not in men (Fig. 5). SHBG was further positively correlated with total oestradiol in pre-menopausal women and also more prominently in post-menopausal women with current HRT use, but very weakly with free oestradiol. Albumin was correlated inversely with oestradiol in both sexes, but most prominently in post-menopausal women with current HRT use. Both total and free testosterone were correlated positively with total and free oestradiol in pre-menopausal women and in men, while there was an inverse correlation between total oestradiol and free testosterone in post-menopausal women with current HRT use. The total and free fractions of each sex steroid were correlated very strongly positively with each other, but less for testosterone in men compared to women (Fig. 5).

## Discussion

Our study has shown prominent positive associations of SHBG with HI, in addition to inverse associations with ABSI and BMI, with lowest levels for “apple” and highest for “pear” phenotype in both sexes and for all BMI categories, but there was only a predominant positive association with HI in obese men. Albumin was associated inversely with HI and BMI in both sexes, but also inversely with ABSI in men, with the lowest levels for “wide” and highest for “slim” phenotype in obese men. In men, total testosterone was associated inversely with ABSI, while free testosterone was associated inversely with ABSI and HI (lowest for “wide” and highest for “slim” phenotype), independent of inverse associations with BMI, which were more prominent for total testosterone. In women, total testosterone was associated weakly inversely with ABSI, while free testosterone was associated positively with ABSI and inversely with HI (lowest for “pear” and highest for “apple” phenotype), and associations with BMI were positive and more prominent for free testosterone. Total oestradiol was associated positively with BMI and with HI in obese men and post-menopausal women never using HRT, but inversely with BMI and with ABSI in obese pre-menopausal women.

The associations of sex steroids and SHBG with BMI are well described and are in agreement with our findings^4–6,8,18,19^, including the unusual inverse association of BMI with total but not with free oestradiol in pre-menopausal women^6,7^. Although associations with waist circumference and WHR have also been reported and are generally directionally consistent with the associations with BMI^4–6,8^, our study justifies the use of allometric indices in order to avoid bias when examining associations with body shape independent of body size. Notably, adjustment for BMI in previous studies had abolished associations of sex steroids and SHBG with waist circumference in pre-menopausal women^7^, and with hip circumference in post-menopausal women^5^, when we have demonstrated associations based on ABSI and HI, independent of associations with BMI.

Considering potential mechanistic explanations, the strong association of SHBG with both waist and hip size in opposite directions would be compatible with an association of SHBG with metabolic dysfunction, which is also related to body shape^2^. SHBG has indeed shown an inverse association with insulin resistance^20^ and shows an association pattern with body shape resembling in inverse that of VAT^11^. VAT, however, was associated positively with ABSI as well as inversely with HI in obese men, while SHBG, similarly to alanine aminotransferase (ALT), high-density and low-density lipoprotein cholesterol, apolipoproteins A1 and B, and triglycerides (examined separately in UK Biobank^21^), was associated predominantly with HI. Notably, total oestradiol, similarly to SHBG, was also associated positively with HI in obese men, suggesting that higher circulating oestradiol at obese BMI may attenuate the associations of SHBG, lipid-related biomarkers, and ALT with waist size. Such a mechanism, however, appears specific to men, as SHBG remained associated inversely with ABSI, as well as positively with HI in obese post-menopausal women who have never used HRT, despite the predominant positive association with HI, similar to men. Nevertheless, the positive association of total oestradiol with HI observed in our study is compatible with subcutaneous and specifically gluteofemoral fat representing a major source of circulating oestradiol both in obese men and in obese post-menopausal women. In men, some 80% of oestradiol are indeed estimated to originate from peripheral aromatisation^22^. In women, a higher aromatase expression in subcutaneous adipose tissue from buttocks compared to abdomen has previously been reported, with an overall increase of aromatase expression with age in all sites^23^. Aromatase expression and conversion of oestrone to oestradiol in women are also higher in the abdominal subcutaneous adipose tissue (ASAT) than in VAT^24,25^. Moreover, we have previously shown for UK Biobank that subcutaneous fat depots, both gluteofemoral fat and ASAT, were associated positively with HI in obese men and women^11^, similarly to total oestradiol. Some small-size intervention studies, however, have suggested that in post-menopausal women it is the central regulation and not the peripheral synthesis that has a dominant role for circulating sex steroid levels. This conclusion was based on similar reductions of circulating total oestradiol and increases in SHBG achieved with weight-reduction interventions, despite a larger loss of fat mass following physical exercise compared to diet, which was additionally accompanied with lean mass loss^26^. In accordance with a central regulation of oestrogens is also the inverse association of total oestradiol with ABSI in obese pre-menopausal women, which is compatible with oestrogen deficiency contributing to higher VAT and hence larger waist size^27^.

Further on potential mechanistic explanations, the inverse association of total testosterone with waist size may reflect the direct influence of testosterone on body shape, as testosterone administration in an intervention study resulted in ABSI reduction^28^. Compatible with the anabolic function of testosterone, ABSI was similarly inversely associated with lean mass^11^. It is interesting, however, that it was total and not free testosterone, which is expected to be biologically active, that matched lean mass. An alternative explanation, therefore, may be a common factor such as glucocorticoids, which in excess lead to visceral obesity, low testosterone levels, and muscle atrophy^29–31^. Glucocorticoids can inhibit gonadal steroid production at all levels of the hypothalamus–pituitary–gonadal axis^32,33^, and contribute to Leiding cell apoptosis^34^. At lower glucocorticoid levels, Leiding cells are protected from glucocorticoids by 11β-hydroxysteroid-dehydrogenase (11βHSD1), acting as oxidase and generating NADPH, which is utilised by 17β-HSD3 in the conversion of androstenedione to testosterone^32^. This defence system, however, is overwhelmed in glucocorticoid excess.

Our findings of higher SHBG and lower free testosterone with older age in men are in agreement with previous studies using immunoassays or mass-spectrometric measurements of testosterone^18,19,35^. A possible explanation could be that the higher SHBG “traps” testosterone and the corresponding reduction in the free testosterone fraction cannot be compensated for, because the hypothalamic response to low free testosterone is blunted with age. Such a mechanism would be compatible with studies reporting no increase of luteinizing hormone (LH) with age^18^. Alternatively, LH may increase with age, as reported in other studies^19^, but Leiding cell counts and gonadal testosterone production decline with aging^36^. Circulating total testosterone levels, however, may also be maintained via peripheral conversion of androstenedione by enzymes such as 17βHSD5, which is higher in mononuclear blood cells of older men^37^.

Our findings of a positive correlation of total oestradiol with SHBG and inverse with free testosterone in post-menopausal women currently using HRT are also compatible with previous reports^4,38^. We, however, additionally report lower levels of oestradiol and testosterone in women with past HRT use compared to never users, which may reflect residual effects of HRT use, given that testosterone levels were further lower in current HRT users. It is possible, however, that women ever using HRT have underlying hormonal differences from women never using HRT, which drive their need for HRT use. In accordance with the latter, women from the Northern Finland Birth Cohort 1966 experiencing earlier climacterium at age 46 years were more likely to use HRT and had fewer pregnancies but more metabolic disturbances, higher fat and lower muscle mass compared to pre-climacteric women at the same age, and additionally had lower testosterone levels even at age 31 years^39^. In our study, post-menopausal women with past or current HRT use had experienced menopause at a younger age compared to those never using HRT.

SHBG has traditionally been considered an inert carrier protein, binding sex steroids with higher affinity but lower capacity compared to albumin, with only free steroids exhibiting steroid action^40^. Some scientists, however, emphasise the potential importance of SHBG binding for the regulation of steroid action, especially in sex steroid target tissues such as the breast, endometrium, prostate, or liver, for which SHBG binding to cellular membranes has been described^41^. In our study, the total levels of the main gonadal steroid for each sex, oestradiol in women and testosterone in men, were associated positively with SHBG, in agreement with previous reports^38,42^. This is compatible with a positive feed-back regulation of SHBG by the main gonadal sex steroid, likely aimed at maintaining stable free steroid levels, as the corresponding free fractions were only weakly associated with SHBG in our and previous studies^43^. The sex steroid of the opposite sex did not appear to exert a positive feed-back regulation on SHBG, as free testosterone in women was inversely associated with SHBG, in agreement with previous reports^43^, although we could not examine associations of free oestradiol with SHBG in men. We observed, however, an apparent suppression of albumin by endogenous and exogenous oestrogens in both sexes, which is in agreement with previously reported lower albumin levels in post-menopausal women using oestradiol replacement therapy^44^.

Strengths of our study are the large sample size, the detailed information for covariates, enabling adjustment for major lifestyle and reproductive factors and thus minimising confounding, the reliable anthropometric measurements obtained by trained personnel according to standardised protocols and thus avoiding bias from self-reported values. Compared to previous reports^6,8^, we have used allometric anthropometric indices and have additionally examined associations with total oestradiol in men and post-menopausal women, associations with albumin and hip size, the combined effect of waist and hip size in body shape phenotypes and heterogeneity according to BMI. We have also used QRILC for the imputation of undetected sex steroids, which has shown better performance, with a smaller distortion of the distribution^45^, compared to the previously used half-minimum imputation of left-censored data^6^.

The major limitation of our study is the insufficient sensitivity and specificity of immunoassays based on competitive binding for low levels of sex steroids, resulting not only in undetected levels but also in bias, with over-recovery of testosterone for low levels and under-recovery for high levels compared to mass-spectrometric measurements^46^, and cross-reactivity with adrenal steroids^47^. The dichotomisation of oestradiol has also admittedly resulted in loss of power but was possible because all samples were evaluated relative to the same cut-off, although with the caveat that there may be misclassification for the borderline values. The large sample size, however, was clearly beneficial, as our findings of lower total oestradiol in post-menopausal women without HRT compared to men were in agreement with reports of mass-spectrometric measurements^48^. A further limitation of our study is the lack of accurate measurements of free testosterone. A recent study has shown an allosteric interaction between the two SHBG binding sites for testosterone, thus countering the assumption of equal affinity underlying the law-of-mass-action equations^49^. Nevertheless, a subsequent study comparing methods for free testosterone calculation against equilibrium dialysis with mass-spectrometric measurement has concluded that although the classical law-of-mass-action equations overestimate free testosterone, they are more robust, as the differences between calculated and measured levels remained independent of SHBG, albumin and testosterone levels, which influenced the new allosteric interaction model^43^.

A further limitation of our study is the lack of information for adrenal androgens, especially 11-hydroxy-androstenedione, which is converted peripherally to the potent androgens 11-oxo-testosterone and 11-oxo-dihydrotestosterone^50^ and is a preferred substrate for 17βHDS5, which increases with age in men^37^. Information for oestrone and hydroxylated oestrogen metabolites, which may modify oestrogen actions^51^, was also lacking. Further, due to limited numbers, we could not examine underweight and severe obesity or ethnic variations, which have been reported for sex steroids^52^. Furthermore, it was not possible to examine directly associations of sex steroids with imaging measurements of body composition, as they were obtained several years apart. We could not examine adequately longitudinal associations either, as follow-up samples were available only for a small part of the cohort. A misclassification of oral contraceptives or HRT use is also possible, as the information was based on three questions with discrepancies in the answers, as well as a misclassification of menopausal status, which is usually established over a prolonged period of time and may be obscured by uterine bleeding induced by HRT use. Last, the UK Biobank cohort lacks younger pre-menopausal women and younger men and is not representative of the overall population, with participants leading a healthier lifestyle^53^.

In conclusion, sex steroids and their binding proteins are associated with hip, as well as with waist size, independent of overall body size. Associations with body shape were most prominent for SHBG, positive with hip and inverse with waist size. Total oestradiol was associated positively with hip size, suggesting that subcutaneous fat represents a major source of circulating oestradiol in obese men and post-menopausal women not using HRT. Oestrogens also appeared to stimulate SHBG and to suppress albumin levels. Total testosterone was associated inversely with waist size, most strongly in men. Total testosterone in men and total oestradiol in women were associated positively with SHBG, suggesting a positive feed-back regulation by the main gonadal steroid for each sex, likely aimed at maintaining constant the free sex steroid levels.

## Supplementary Information

Supplementary Information.

## Data Availability

The data supporting the findings of the study are available *to bona fide* researchers upon approval of an application to the UK Biobank (https://www.ukbiobank.ac.uk/researchers/) and a material transfer agreement.

## Abbreviations

ABSI: A body shape index
BMI: Body mass index
CI: Confidence interval
HC: Hip circumference
HI: Hip index
NHANES: National Health and Nutrition Examination Survey
SD: Standard deviation
UK: United Kingdom
VAT: Visceral adipose tissue
WC: Waist circumference
WHR: Waist-to-hip ratio
