# Supplementary material for: Associations of body shape phenotypes with sex steroids and their binding proteins in the UK Biobank cohort
Source: Sci Rep. 2022 Jun 24;12:10774. doi: 10.1038/s41598-022-14439-9 (PMC9232606; doi:10.1038/s41598-022-14439-9)
Supplement: Supplementary file 1 — Supplementary Information. [file 41598_2022_14439_MOESM1_ESM.pdf]

Associations of body shape phenotypes with sex steroids and their binding proteins in the UK Biobank cohort

Sofia Christakoudi, Elio Riboli, Evangelos Evangelou, Konstantinos K. Tsilidis

Supplementary Methods

Imputation of undetectable biomarker levels 2

Calculation of free sex steroid fractions 4

Definition of covariates 4

Supplementary Tables

Supplementary Table S1 Classification of self-reported steroid-related drugs 10

Supplementary Table S2 Characteristics of study participants 13

Supplementary Table S3 Associations of sex steroids and their binding proteins with age at enrolment 16

Supplementary Table S4 Associations of sex steroids and their binding proteins with body size and body shape indices (continuous) 17

Supplementary Table S5 Associations of sex steroids and their binding proteins with body mass index (categorical) 19

Supplementary Table S6 Associations of sex steroids and their binding proteins with body shape phenotypes (categorical) 21

Supplementary Figures

Supplementary Figure S1 Flow diagram of UK Biobank participants included in the study 27

Supplementary Figure S2 Associations of body shape indices with sex steroids and their binding proteins: heterogeneity by BMI 29

References **31**

Supplementary Methods

Imputation of undetectable biomarker levels

Missingness for biomarkers was determined based on Field [30806-0.0] "*Oestradiol reportability*", Field [30856-0.0] "*Testosterone reportability*", Field [30836-0.0] "*SHBG reportability*" and Field [30606-0.0] "*Albumin reportability*". Missingness per biomarker is summarised in the Table below (number, % from total in group):

	Low (Undetected)	Reported	High	Available	Missing
**Men (overall) n=179,902**					
SHBG	2	165,288 (91.9)	-	165,290 (91.9)	14,612 (8.1)
Albumin	2	165,867 (92.2)	-	165,869 (92.2)	14,033 (7.8)
Testosterone	13	179,010 (99.5)	10	179,033 (99.5)	869 (0.5)
Oestradiol	153,225 (85.2)	15,048 (8.4)	-	168,273 (93.5)	11,629 (6.5)
**Men (<55 years) n=69,378**					
SHBG	2	63,751 (91.9)	-	63,753 (91.9)	5,625 (8.1)
Albumin	1	63,970 (92.2)	-	63,971 (92.2)	5,407 (7.8)
Testosterone	1	69,035 (99.5)	6	69,042 (99.5)	336 (0.5)
Oestradiol	58,832 (84.8)	6,003 (8.7)	-	64,835 (93.5)	4,543 (6.5)
**Men (≥55 years) n=110,524**					
SHBG	-	101,537 (91.9)	-	101,537 (91.9)	8,987 (8.1)
Albumin	1	101,897 (92.2)	-	101,898 (92.2)	8,626 (7.8)
Testosterone	12	109,975 (99.5)	4	109,991 (99.5)	533 (0.5)
Oestradiol	94,393 (85.4)	9,045 (8.2)	-	103,438 (93.6)	7,086 (6.4)
**Women (overall) n=207,444**					
SHBG	4	186,892 (90.1)	566 (0.3)	187,462 (90.4)	19,982 (9.6)
Albumin	6	188,196 (90.7)	-	188,202 (90.7)	19,242 (9.3)
Testosterone	31,352 (15.1)	175,259 (84.5)	-	206,611 (99.6)	833 (0.4)
Oestradiol	145,257 (70.0)	48,835 (23.5)	-	194,092 (93.6)	13,352 (6.4)
**Women Pre-MP n=40,956**					
SHBG	1	36,828 (89.9)	19	36,848 (90.0)	4,108 (10.0)
Albumin	2	36,956 (90.2)	-	36,958 (90.2)	3,998 (9.8)
Testosterone	2,510 (6.1)	38,314 (93.5)	-	40,824 (99.7)	132 (0.3)
Oestradiol	8,678 (21.2)	29,646 (72.4)	-	38,324 (93.6)	2,632 (6.4)
**Women Post-MP Never-HRT n=63,134**					
SHBG	1	57,172 (90.6)	12	57,185 (90.6)	5,949 (9.4)
Albumin	1	57,403 (90.9)	-	57,404 (90.9)	5730 (9.1)
Testosterone	8,838 (14.0)	54,026 (85.6)	-	62,864 (99.6)	270 (0.4)
Oestradiol	57,064 (90.4)	2,032 (3.2)	-	59,087 (93.6)	4047 (6.4)
**Women Post-MP Past-HRT n=54,591**					
SHBG	-	49,441 (90.6)	6	49,447 (90.6)	5,144 (9.4)
Albumin	-	49,625 (90.9)	-	49,625 (90.9)	4,966 (9.1)
Testosterone	10,994 (20.1)	43,364 (79.4)	-	54,358 (99.6)	233 (0.4)
Oestradiol	50,123 (91.8)	980 (1.8)	-	51,103 (93.6)	3,488 (6.4)
**Women Post-MP Current-HRT n=10,722**					
SHBG	-	9,501 (88.6)	154 (1.4)	9,655 (90.0)	1,067 (10.0)
Albumin	1	9,730 (90.7)	-	9,731 (90.8)	991 (9.2)
Testosterone	2,777 (25.9)	7,904 (73.7)	-	10,681 (99.6)	41 (0.4)
Oestradiol	6,399 (59.7)	3,628 (33.8)	-	10,027 (93.5)	695 (6.5)

Note that "missing" are those for which measurement was not attempted, while "undetected" are those for which measurement was attempted but the level was below the limit of detection.

Values flagged with code 1: "*Reportable at assay and after aliquot correction, if attempted*" were used as provided. Values flagged with code 2: "*Reportable at assay but not reportable after any corrections (too low)*" or code 4: "*Not reportable at assay (too low)*" were considered below the lower limit of detection and were replaced with the lower detected level for testosterone in men and for SHBG and Albumin in both sexes, as numbers were limited, but for testosterone in women and for oestradiol in pre-menopausal women were imputed with quantile regression imputation of left-censored data (QRILC) (**imputeLCMD** v2.0 package in R), after log-transformation. Values flagged with code 3: "*Reportable at assay but not reportable after any corrections (too high)*" or code 5: "*Not reportable at assay (too high)*" were considered higher than the upper limit of reportability and were replaced with the highest detected value. Values for samples with missing code in the above fields were considered missing and the corresponding participants were excluded from the analysis of the corresponding biomarker.

The lowest detected values were 0.39 nmol/L for SHBG, 18.87 g/L for Albumin, 0.35 nmol/L for testosterone and 175 pmol/L for oestradiol. The highest reported values were 241.58 nmol/L for SHBG and 53.136 nmol/L for testosterone.

Testosterone and oestradiol levels were log-transformed prior to imputation with function **impute.QRILC**. Testosterone was imputed in all women with attempted measurements of testosterone as one group, with tuning parameter sigma for the standard deviation set to 3, to account for deviations from the Gaussian distribution. Oestradiol was imputed in women from the Pre-MP group with attempted measurements of oestradiol as one group, with tuning parameter sigma set to 1.5. Oestradiol was not imputed for the Post-MP Current-HRT group, as less than half of the samples had detectable levels, excluding the peak of the distribution.

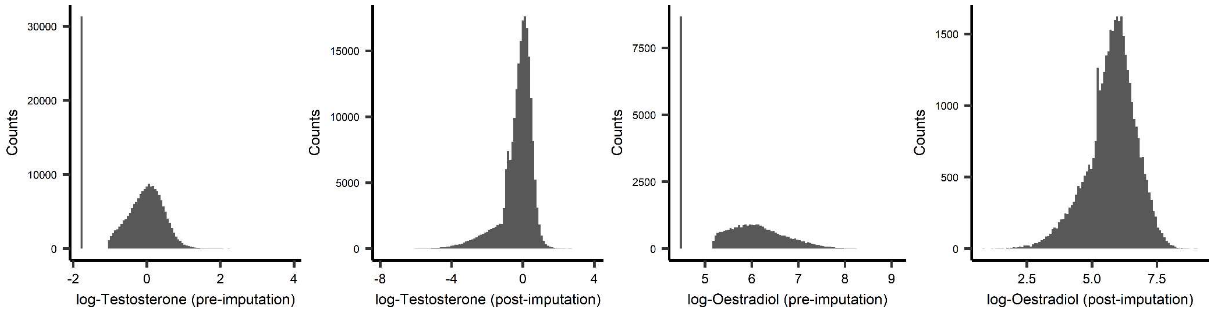

The histograms above show the distributions of log-transformed sex steroids prior to and post imputation (the counts for low undetectable levels are shown as a single separate bar in the left-hand side of the pre-imputation plots). For the statistical analyses, the log-transformed values post imputation were converted to sex-specific z-scores (value minus mean divided by the standard deviation). Imputed values were exponentiated back to linear scale for plots and summaries of testosterone and oestradiol levels.

Calculation of free sex steroid fractions

Free testosterone and oestradiol were calculated according to the law-of-mass-action equations proposed by Sodergard *et al.* [ref. 16].

$$[fS] = \{-b + \sqrt{(b^2 + 4a * S)}\} / 2a$$

$$a = k_{albumin} + k_{SHBG} + (k_{albumin} * k_{SHBG}) * ([SHBG] + [albumin] – [S])$$

$$b = 1 + k_{SHBG} * [SHBG] + k_{albumin} * [albumin] – (k_{albumin} + k_{SHBG}) * [S]$$

where k_{SHBG} and $k_{albumin}$ were the association constants of sex steroids with SHBG and albumin, correspondingly, with values $k_{SHBG} = 5.97*10^8$ L/mol and $k_{albumin} = 4.06*10^4$ L/mol for testosterone and $k_{SHBG} = 3.14*10^8$ L/mol and $k_{albumin} = 4.21*10^4$ L/mol for oestradiol. [S] (measured) and [fS] (calculated) were, correspondingly, the concentrations of the total and the free sex steroid of interest (testosterone or oestradiol) in mol/L. [SHBG] was the measured concentration of SHBG in mol/L. Conversion from nmol/L to mol/L involved a division by 10^9. Conversion from mol/L involved a multiplication with 10^9. [albumin] was the measured concentration of albumin, converted from g/L to mol/L by division with the molecular weight 69,000 Da.

Definition of covariates

Age at enrolment (used on a continuous scale with 5 years increment), region of the assessment centre, weight change during the last year preceding enrolment, smoking status, physical activity, alcohol consumption, prevalent and incident cancers, and deaths (used for exclusions) were defined as previously described [ref. 3].

Fasting time was based on Field [74-0.0] "*Fasting time*". Three categories were defined as follows: 0-2 hours, 3-4 hours, 5 or more hours. Samples with unknown fasting time were assigned the sex-specific median category 3-4 hours. On a continuous scale, missing values were replaced with the sex-specific median (3 hours for both sexes).

Time of blood collection was based on Field [3166-0.0] "*Time blood sample collected*". Time in hours was defined as: (Hours * 3600 + Minutes * 60 + Seconds) / 3600. Three categories were defined, separating the day in three parts: Morning (<12 o'clock, first at 8:25), Afternoon (≥12 to <16 o'clock), Evening (≥16 o'clock, last at 21:15). Samples with unknown time of collection were assigned the sex-specific median category, Afternoon. On a continuous scale, missing values were replaced with the sex-specific median (14.38 for women, 14.48 for men).

Townsend deprivation index, based on Field [189-0.0] "*Townsend deprivation index at recruitment*", was used as an indicator of socioeconomic status. Missing values were replaced with the sex-specific median: -2.295 for women and -2.271 for men. The tertile boundaries for the dataset used in this study were: -3.231 and -0.908 for women and -3.235 and -0.822 for men.

Hand grip strength, used as an indicator of muscle functionality, was defined as in [ref. 11]. Missing values were replaced with the sex-specific median (26 for women, 42 for men).

Cholesterol lowering drugs use was based on Fields [6153-0.0/3] "*Medication for cholesterol, blood pressure, diabetes, or take exogenous hormones*", Question: "*Do you regularly take any of the following medications? (You can select more than one answer)"*. Participants providing Answer 1 "*Cholesterol lowering medication*" were assigned to category Yes and those providing answer -7 "None of the above" or only answers 2 "Blood pressure medication", or 3 "Insulin", or in women 4 "Hormone replacement therapy", or 5 "Oral contraceptive pill or minipill" were assigned to category No. For the remaining participants, this information was considered missing and for them was used the sex-specific median category, No for both sexes.

Oophorectomy (bilateral) was used to define menopausal status and was based on Field: [2834-0.0] "*Bilateral oophorectomy (both ovaries removed)*"; Question: "*Have you had BOTH ovaries removed?*"; Answer 1: "*Yes*" OR an answer to Fields [20004-0.0/31] "*Operation code (self-reported operation)*" including code: 1355 "*bilateral oophorectomy*".

Hysterectomy was used to define menopausal status and was based on Field: [3591-0.0] "*Ever had hysterectomy (womb removed)*"; Question: "*Have you had a hysterectomy (womb removed)?";* Answer 1: "*Yes*" OR an answer to Fields [20004-0.0/31] "*Operation code (self-reported operation)*" including codes: 1357 "*hysterectomy*", 1358 "*hysterectomy with oophorectomy*", or 1359 "*hysterectomy with cervical sparing*".

Menopausal status (MP) was defined in three categories: Post-menopausal were classified women with bilateral oophorectomy (n=21,990 in the total UK Biobank cohort) OR with self-reported post-menopausal status from Field [2724-0.0] "*Had menopause*"; Question: "*Have you had your menopause (periods stopped)?*"; Answer 1: "Yes" (additional n=156,073); Pre-menopausal were classified women who had not been defined as post-menopausal above AND had reported pre-menopausal status with Answer 0: "No" to Field [2724-0.0] AND had not reported hysterectomy (n=63,631); Undetermined included the remaining women (n=31,659).

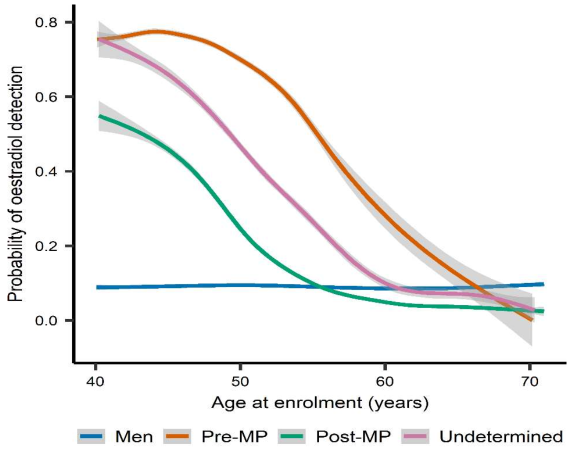

Oestrogen levels were lower for older age in all subgroups by menopausal status and women in the undetermined group showed intermediate oestradiol levels at all ages (plot above). We, therefore, excluded women with undetermined menopausal status and set age restrictions for subgroups by menopausal status (see the definitions of subgroups in women below).

Time of menstrual period was defined for pre-menopausal women and was based on Field [3700-0.0] "*Time since last menstrual period*"; Question: "*How many days since your last menstrual period?"*. Answers *-1 "Do not know"* and *-3 "Prefer not to answer"* were considered missing values. Seven groups were defined as follows: Early follicular (days 0-5, n=15,483 in the total UK Biobank cohort); Late follicular (days 6-10, n=10,507); Mid-cycle (days 11-14, n=7,757); Early luteal (days 15-18, n=5,171); Mid-luteal (days 19-24, n=8,870); Late luteal (days 25-40, n=6,114); Undetermined (days ≥41 or missing, n=9,729). These divisions are based on [ref. 6], with the exception that we have introduced an upper limit for the late luteal phase and have classified women further away from their previous period than this limit as undetermined.

Age at the last live birth was defined as in [ref. 3]. Category No live births included n=51,097 women in the total UK Biobank cohort; category 30 years included n=104,948 women, category ≥ 30 years included n=115,649 women. Women with missing information (n=1,659) were assigned to the median category in the study dataset, <30 years.

Hormone replacement therapy (HRT) use was determined for women based on all relevant fields. Three groups were defined based on Field [2814-0.0] "*Ever used hormone-replacement therapy (HRT)*"; Question: "*Have you ever used hormone replacement therapy (HRT)?*" as follows: Current use – Answer 1: "Yes" in Field [2814-0.0] AND Answer -11: "*Still taking HRT"* in Field [3546-0.0] "*Age last used hormone-replacement therapy (HRT)*" Question: *"How old were you when you last used HRT?"* (n=16,446 in the total UK Biobank cohort); Former use – Answer 1: "Yes" in Field [2814-0.0] with any other answer (not -11) or missing in Field [3546-0.0] (n=87,461); Never use – Answer 0: "*No*" in Field [2814-0.0] (n=167,872). Additional information was derived from Fields [6153-0.0/3] "*Medication for cholesterol, blood pressure, diabetes, or take exogenous hormones*", Question: "*Do you regularly take any of the following medications? (You can select more than one answer)"*. Women providing Answer 4 "*Hormone replacement therapy*", classified in the previous step as Never user, Former user or had missing information for HRT use, were re-classified to Current use (additional n=3,998). Further information was derived from Fields [2003-0.0/47] "Treatment/medication code". Women receiving medications containing compounds with oestrogenic activity, individually or in combination with progestins (except oral contraceptives, see list in Supplementary Table S1) (additional n=2,462), as well as post-menopausal women or women with undetermined menopausal status receiving medications containing only progestins (additional n=374), and classified in the previous steps as Never user, Past user or had missing information for HRT use, were re-classified to Current use. The final count in the total UK Biobank cohort was: Never user (n=166,468), Former user (n=82,132) and Current user (n=23,280). Women with missing information for HRT use following the above assignments (n=1,473) were excluded from this study.

HRT type (Current-HRT) was defined as Oestrogen only if post-menopausal women with current HRT use had reported in Fields [2003-0.0/47] “Treatment/medication code” use of medication containing only oestrogen and not use of combined or progestin-only medication (n=10,244 in the total UK Biobank cohort) (see Supplementary Table S1 for list of drugs). Women were assigned to category Combined or progestin-only if they had reported in Fields [2003-0.0/47] use of medications containing oestrogens and progestins (as individual or as combined medications), or only progestins (n=7,664). The information was considered missing for women not assigned to the above categories (n=5,372).

Oral contraceptives use was determined for women based on all relevant fields. Three groups were defined based on Field [2784-0.0] “*Ever taken oral contraceptive pill*”; Question: "*Have you ever taken the contraceptive pill? (include the 'mini-pill')*" as follows: Current use – Answer 1: “Yes” in Field [2784-0.0] AND Answer -11: “*Still taking the pill”* in Field [2804-0.0] “*Age when last used oral contraceptive pill*” Question: *"How old were you when you last used the contraceptive pill?"* (n=4,918 in the total UK Biobank cohort); Former use – Answer 1: “Yes” in Field [2784-0.0] with any other answer (not -11) or missing in Field [2804-0.0] (n=215,493); Never use – Answer 0: “*No*” in Field [2784-0.0] (n=51,525). Additional information was derived from Fields [6153-0.0/3], as for HRT use. Women providing Answer 5 “*Oral contraceptive pill or minipill*”, classified in the previous step as Never user, Past user or with missing information for oral contraceptives use, were re-classified to Current use (additional n=2,355). Further information was derived from Fields [2003-0.0/47], as for HRT use. Women receiving contraceptives (n=3,130), as well as pre-menopausal women receiving medications containing only progestins (n=442) (see Supplementary Table S1 for a list of medications), and classified in the previous steps as Never user, Past user or with missing information for oral contraceptives use, were re-classified to Current use. Note that although some of the medications included in Fields [2003-0.0/47] represent intrauterine steroid containing devices, we have retained “oral” in the naming of this variable as the main information was derived from the fields enquiring about use of oral contraceptives. The final count in the total UK Biobank cohort was Never use (n=51,209), Former use (n=209,907) and Current use (n=10,845). Women with missing information for oral contraceptive use following the above assignments (n=1,392) were excluded from this study.

Subgroups of women were defined based on self-reported menopausal status, with restriction for age and use of oral contraceptives and HRT (see definitions of these variables above and Supplementary Figure S1 for illustration). Pre-MP – included pre-menopausal women younger than 55 years of age, not using oral contraceptives or cholesterol lowering drugs at enrolment and never using HRT. Only a small number of pre-menopausal women younger than 55 years and not using oral contraceptives at enrolment had used HRT in the past (n=650) or were using HRT at enrolment (n=684), which precluded examining these as separate subgroups. The number of pre-menopausal women never HRT users using cholesterol lowering drugs at enrolment was also low (n=862) and they were excluded from the subgroup; Post-MP Never-HRT – included post-menopausal women aged 50 years or older who have never used HRT and were not using oral contraceptives at enrolment; Post-MP Former-HRT – included post-menopausal women aged 50

years or older who had used HRT in the past and were not using oral contraceptives at enrolment; Post-MP Current-HRT – included post-menopausal women aged 50 years or older who were using HRT but not oral contraceptives at enrolment. The transitional period (≥50 to <55 years) included 9,118 pre-menopausal women (18.2% of all pre-menopausal women) and 17,964 post-menopausal women (13.4% of all post-menopausal women). Women with self-reported pre-menopausal status but aged 55 years or older (n=1,330, 2.7 % of pre-menopausal women) and women with self-reported post-menopausal status but younger than 50 years (n=4,405, 3.3 % of post-menopausal women) were excluded from the subgroup analyses, as they were a small proportion and unrepresentative of the subgroup of women with the corresponding menopausal status.

Time since stopped HRT use (past HRT use) was derived for the study dataset by subtracting Field [3546-0.0] "*Age last used hormone-replacement therapy (HRT)*"; Question: "*How old were you when you last used HRT?*" from Age at enrolment. Answers *-1 "Do not know"* and *-3 "Prefer not to answer"* were considered missing values. To accommodate the large missingness, three categories were defined as follows: < 7 years (close to the median of the study dataset for women with past HRT use) (n=29,004 in the study dataset), ≥ 7 years (n=26,508), or Unknown (n=7,462).

Duration of HRT use (past HRT use) was derived for the study dataset by subtracting Field [3536-0.0] "*Age started hormone-replacement therapy (HRT)*"; Question: "*How old were you when you first used HRT?*" from Field [3546-0.0] "*Age last used hormone-replacement therapy (HRT)*". Answers *-1 "Do not know"* and *-3 "Prefer not to answer"* were considered missing values. To accommodate the large missingness, three categories were defined as follows: < 6 years (close to the median of the study dataset for women with past HRT use) (n=28,019 in the study dataset), ≥ 6 years (n=25,888), or Unknown duration (n=9,067).

Duration of HRT use (current HRT use) was derived for the study dataset by subtracting Field [3536-0.0] "*Age started hormone-replacement therapy (HRT)*" from Age at enrolment. To accommodate the large missingness, three categories were defined as follows: < 11 years (close to the median of the study dataset for women with current HRT use) (n=9,080 in the study dataset), ≥ 11 years (n=7,179), or Unknown duration (n=1,653).

HRT use and duration was used as covariate in models for women overall and was defined for the study dataset as a combined variable with seven categories as follows: Never use (n=126,558 in the study dataset); Past use <6 years (n=28,019); Past user ≥6 years (n=25,888); Past use Unknown duration (n=9,067); Current use <11 years (n=9,080); Current use ≥11 years (n=7,179); Current use Unknown duration (n=1,653).

Time since stopped oral contraceptives use (pre-menopausal, past use) was derived for the study dataset by subtracting Field [2804-0.0] "*Age when last used oral contraceptive pill*"; Question: "*How old were you when you last used the contraceptive pill?*" from Age at enrolment. To accommodate the large missingness, four categories were defined as follows: < 10 years (n=8,382 in the study dataset), ≥ 10 to <20 years (n=16,232), ≥ 20 years (n=12,967), or Unknown

time (n=2,288). The cut-offs correspond to the tertile boundaries for pre-menopausal women with past OC use rounded to ten years.

Time since stopped oral contraceptives use (post-menopausal or undetermined, past use) was derived as for pre-menopausal women, but the four categories were defined as follows: < 20 years (n=21,594 for the study dataset), ≥ 20 to <30 years (n=42,037), ≥ 30 years (n=45,157), or Unknown time (n=14,334). The cut-offs correspond to the tertile boundaries for women with post-menopausal or undetermined status and past oral contraceptives, use rounded to ten years.

Oral contraceptives use with time since stopped was defined for the study dataset as a combined variable with six categories as follows: Never use (n=35,855 for the study dataset); Past use <10 (or<20) years (n=29,976); Past use ≥10 to <20 (or ≥20 to <30) years (n=58,269); Past use ≥20 (or ≥30) years (n=58,124), Past use Unknown time (n=16,632); Current use (n=8,588). The alternative cut-offs correspond to pre-menopausal women (or women with post-menopausal or undetermined status).

Age at menarche was based on Field [2714-0.0] "*Age when periods started (menarche)*"; Question: "*How old were you when your periods started?".* Answers *-1 "Do not know"* and *-3 "Prefer not to answer"* were considered missing values.

Age at menopause was defined for post-menopausal women and was based on Field [3581-0.0] "*Age at menopause (last menstrual period)*"; Question: "*How old were you when your periods stopped?"* OR Field [3882-0.0] "*Age at bilateral oophorectomy (both ovaries removed)*"; Question: "*How old were you when you had BOTH ovaries removed?"*, using the lesser of the two ages when both were available. Answers *-1 "Do not know"* and *-3 "Prefer not to answer"* were considered missing values.

Supplementary Table S1 Classification of self-reported steroid-related drugs

Code	Name	Code	Name
**Part A: Included in the study**			
**Oestrogen: assigned to HRT-Oestrogen-only**			
1140857690	oestradiol 25mg implant 36 week	1140884624	fosfestrol
1140857700	oestradiol 1mg/1ml injection	1140909848	diethylstilbestrol
1140857706	oestriol 250micrograms tablet	1140909906	estropipate
1140857708	quinestradol	1140911708	estring 2mg(7.5micrograms/24hours) vaginal ring
1140857714	quinestrol	1140916790	evorel 25 patch
1140857716	estrovis 4mg tablet	1140917448	oestradiol 1.25g/dose gel
1140868372	climaval 1mg tablet	1140917450	oestrogel 1.25g gel
1140868400	oestriol product	1140922804	premique 0.625mg/5mg tablet
1140868406	conjugated oestrogens	1140922806	premique cycle 10mg tablet
1140868408	premarin 625micrograms tablet	1140923598	fematrix 40 patch
1140868420	piperazine oestrone sulphate	1140923738	femseven 50 patch
1140868422	harmogen 1.5mg tablet	1140923852	elleste-solo 1mg tablet
1140868446	ethinyloestradiol	1140923914	progynova ts 50micrograms patch
1140868456	oestradiol product	1140926592	estraderm mx 25 patch
1140868458	hormonin tablet	1140928878	zumenon 1mg tablet
1140868472	vagifem 25mcg pessary	1141157404	ethinyloestradiol product
1140868722	cyclofenil	1141166196	etonogestrel
1140868724	rehibin 100mg tablet	1141167206	oestrogel 0.06% gel
1140869032	dienoestrol	1141177158	adgyn estro 2mg tablet
1140869036	ovestin 0.1% vaginal cream	1141180580	progynova ts 50 50micrograms patch
1140869046	tampovagan pessary	1141180988	dienestrol
1140869524	estracyt 140mg capsule	1141181218	ethinylestradiol product
1140870062	estradurin 40mg injection (pdr for recon)+diluent	1141181220	ethinylestradiol
1140870070	stilboestrol	1141181594	estriol product
1140870186	oestrifen 10mg tablet	1141181700	estradiol product
1140879554	estramustine	1141192440	piperazine estrone sulphate
1140883014	ortho-dienoestrol 0.01% cream	1141202030	estradot 25micrograms patch
1140884622	oestrogen product		
**HRT: assigned to HRT-Combined/Progestin-only**			
1140857636	prempak 0.625 tablet	1140926430	climesse tablet
1140857932	hormofemin 0.025% cream	1140926686	femapak 40 patch+tablet
1140864196	climagest 1mg tablet	1141151368	sandrena 0.5mg gel
1140868460	progynova 1mg tablet	1141151718	evorel conti patch
1140868470	estrapak 50micrograms/1mg patch+tablet	1141152228	elleste-solo mx 40 patch
1140868482	tibolone	1141156644	elleste duet conti tablet
1140868508	cyclo-progynova 1mg tablet	1141168324	oestradiol+norethisterone acetate 1mg/0.5mg tablet
1140868512	syntex menophase tablet	1141168574	raloxifene hydrochloride
1140868514	trisequens tablet	1141168578	evista 60mg tablet
1140868518	nuvelle tablet	1141172436	indivina 1mg/2.5mg tablet
1140868520	estracombi tts patch	1141177226	adgyn combi 2mg tablet
1140869034	ortho-gynest 500micrograms pessary	1141179820	drospirenone
1140882946	livial 2.5mg tablet	1141180766	novofem tablet
1140884626	mestranol	1141181818	estradiol+norethisterone acetate 1mg/0.5mg tablet
1140921088	tridestra tablet	1141190580	conjugated oestrogens 0.3mg / medroxyprogesterone 1.5mg tab
1140922562	femoston 1/10 tablet		
**Contraceptive: assigned to OC**			
1140857650	controvlar tablet	1140869348	ethinyloestradiol+norgestimate 35mcg/250mcg tablet
1140857986	anovlar-21 tablet	1140869352	norinyl-1 tablet
1140857988	gynovalr-21 tablet	1140869354	ortho-novin 1/50 tablet
1140869162	marvelon tablet	1140869356	mestranol+norethisterone 50micrograms/1mg tablet
1140869164	mercilon tablet	1140869362	femulen tablet
1140869166	ethinyloestradiol+desogestrel 20mcg/150mcg tablet	1140869366	levonorgestrel
1140869172	ethinyloestradiol+ethynodiol diacetate 30mcg/2mg tablet	1140869368	microval tablet
1140869174	eugynon 30 tablet	1140869370	norgeston tablet

Code	Name	Code	Name
**Contraceptive: assigned to OC** *(continued)*			
1140869176	logynon tablet	1140876638	cyproterone acetate+ethinyloestradiol
1140869180	microgynon 30 tablet	1140880234	dianette tablet
1140869184	ovran 30 tablet	1140883162	combined oral contraceptive product
1140869186	ovranette tablet	1140917056	kliofem tablet
1140869248	ethinyloestradiol+levonorgestrel 30mcg/150mcg tablet	1140921814	mirena 52mg intrauterine system
1140869254	binovum tablet	1140921822	mirena 20mcg/24hrs intrauterine system
1140869256	brevinor tablet	1141166200	implanon 68mg subdermal implant
1140869258	neocon 1/35 tablet	1141166366	ethinyloestradiol+gestodene 20micrograms/75micrograms tablet
1140869260	norimin tablet	1141166368	femodette tablet
1140869262	ovysmen tablet	1141168326	kliovance 1mg/0.5mg tablet
1140869264	synphase tablet	1141172722	levonelle 750micrograms tablet
1140869266	trinovum tablet	1141179822	ethinylestradiol+drospirenone 30micrograms/3mg tablet
1140869272	neogest tablet	1141179824	yasmin tablet
1140869276	micronor tablet	1141181204	ethinylestradiol+norgestimate 35mcg/250mcg tablet
1140869282	noristerat 200mg/1ml oily injection	1141181240	ethinylestradiol+levonorgestrel 30mcg/150mcg tablet
1140869324	loestrin 20 tablet	1141181286	ethinylestradiol+desogestrel 20mcg/150mcg tablet
1140869328	ethinyloestradiol+norethisterone acetate 20mcg/1mg tablet	1141181298	ethinylestradiol+norethisterone acetate 20mcg/1mg tablet
1140869332	minulet tablet	1141181306	ethinylestradiol+gestodene 20micrograms/75micrograms tablet
1140869334	femodene tablet	1141182794	desogestrel product
1140869338	tri-minulet tablet	1141182800	cerazette 75micrograms tablet
1140869340	triadene tablet	1141192344	cyproterone acetate+ethinylestradiol
1140869346	cilest tablet	1141192874	ethinylestradiol+norelgestromin 600mcg/6mg transdermal patch
**Progestin: assigned to OC (pre-menopausal) or HRT-Combined/Progestin-only (post-menopausal or undetermined)**			
1140857620	depo-provera 50mg/1ml injection	1140868588	progesterone product
1140857628	gestone 10mg/1ml injection	1140868590	cyclogest 200mg suppository
1140857912	desogestrel	1140869270	medroxyprogesterone
1140857918	lynoestrenol	1140869278	noriday tablet
1140857990	minovlar tablet	1140869360	ethynodiol diacetate
1140858324	medroxyprogest 80mg/ml suspension 100ml	1140870144	farlutal 100mg tablet
1140864232	provera 2.5mg tablet	1140870232	megace 40mg tablet
1140868330	progesic 200mg tablet	1140884686	gestronol
1140868488	allyloestrenol	1140884688	hydroxyprogesterone
1140868490	gestanin 5mg tablet	1140884706	megestrol
1140868494	dydrogesterone	1140910562	gestonorone
1140868496	duphaston 10mg tablet	1141157406	norethisterone product
1140868554	proluton depot 250mg/1ml oily injection	1141157410	levonorgestrel product
1140868580	norethisterone	1141172714	climanor 5mg tablet
1140868584	primolut-n 5mg tablet	1141177150	adgyn medro 5mg tablet
1140868586	utovlan 5mg tablet		
**Part B: Excluded from the study**			
**Anti-oestrogen**			
1140868714	clomid 50mg tablet	1140870182	emblon 10mg tablet
1140868716	serophene 50mg tablet	1140884638	clomiphene
1140870164	tamoxifen	1140927794	fareston 60mg tablet
1140870170	nolvadex 10mg tablet	1141180944	clomifene
1140870176	tamofen 10mg tablet		
**Anti-Progestin**			
1140869012	mifegyne 200mg tablet	1141157302	mifepristone product
1140869112	mifepristone		
**Anti-androgen**			
1140851508	spiroprop tablet	1140870260	cyprostat 50mg tablet
1140866236	spironolactone	1140870274	flutamide
1140866306	spirospare 25mg tablet	1140870278	drogenil 250mg tablet
1140866318	spirolone 25mg tablet	1140884634	cyproterone
1140868524	androcur 50mg tablet		

Code	Name	Code	Name
**Androgen**			
1140857634	mixogen tablet	1140868618	stanozolol
1140857656	methyltestosterone product	1140868620	stromba 5mg tablet
1140857668	virormone-oral 5mg tablet	1140868968	danazol
1140858338	drostanolone propionate	1140868972	danol 100mg capsule
1140864502	testotop tts 15mg transdermal patch	1140868978	gestrinone
1140865136	yohimbine/pemoline/methyltestosterone	1140868982	dimetriose 2.5mg capsule
1140866232	spiroctan-m 200mg/10ml injection	1140884726	nandrolone
1140866312	spiroctan 25mg tablet	1140910674	ethinylnortestosterone
1140868526	mesterolone	1140910802	androstanazol
1140868528	pro-viron 25mg tablet	1140928222	andropatch 2.5mg/24hours transdermal patch
1140868532	testosterone product	1141166354	testoderm 6mg/24hours transdermal patch
1140868534	primoteston depot 250mg/1ml oily injection	1141193272	testogel 50mg gel 5g sachet
1140868536	restandol 40mg capsule	1141195062	striant sr 30mg muco-adhesive buccal tablet
1140868538	sustanon 100 oily injection	1141201718	nebido 1000mg/4ml solution for injection
1140868614	deca-durabolin 25mg/1ml oily injection		
**5α-reductase inhibitor**			
1140868550	finasteride	1141192000	dutasteride
1140868608	proscar 5mg tablet	1141192004	avodart 500micrograms capsule
1141179886	propecia 1mg tablet		
**Steroid synthesis blocker**			
1140868892	trilostane	1140868958	metopirone 250mg capsule
1140868894	modrenal 60mg capsule	1140870242	aminoglutethimide
1140868956	metyrapone	1140870244	orimeten 250mg tablet
**Gonadotropin, gonadotropin releasing hormone, or inhibitor**			
1140863580	hrf-ayerst 100micrograms injection	1140909920	gonadotrophin-releasing hormone product
1140864446	normegon 75iu injection+solvent	1140909922	gnrh - gonadotrophin-releasing hormone product
1140864922	orgafol 75iu injection (pdr for recon)+diluent	1140909924	lh-rh - gonadotrophin-releasing hormone
1140868628	humegon 75iu injection (pdr for recon)+solvent	1140910638	luteal hormone
1140868730	pergonal 75iu injection (pdr for recon)+solvent	1140910640	luteine
1140868882	gonadorelin	1140921100	triptorelin
1140868884	synarel 200micrograms nasal spray	1141157392	buserelin product
1140868936	fertiral 1mg/2ml injection	1141157394	goserelin product
1140868938	hrf 100micrograms injection (pdr for recon)+diluent	1141165318	cetrorelix
1140868942	relefact lh-rh 100micrograms/1ml injection	1141165324	cetrotide 0.25mg injection (pdr for recon)+solvent
1140868984	nafarelin	1141171536	ganirelix
1140870194	goserelin	1141171540	orgalutran 0.25mg/0.5ml prefilled syringe
1140870196	zoladex 3.6mg implant	1141177658	menopur 75iu injection (pdr for recon)+solvent
1140870248	buserelin	1141182558	urofollitropin
1140870252	suprefact 100micrograms nasal spray	1141184648	human luteinising hormone product
1140870284	prostap sr 3.75mg injection (pdr for recon)+diluent+kit	1141184652	lutropin alfa
1140882960	urofollitrophin	1141184654	recombinant human luteinising hormone alfa
1140882962	human menopausal gonadotrophins	1141184712	luveris 75iu injection (pdr for recon)+solvent
1140884544	leuprorelin	1141189772	gonapeptyl depot 3.75mg inj (pdr for recon)+solv p/f syringe
**Glucocorticoids**			
1140857532	cortelan 25mg tablet	1140874896	hydrocortisone
1140857534	oradexon 500micrograms tablet	1140874930	prednisolone
1140865840	predfoam 20mg enema	1140874936	deltacortril enteric 2.5mg e/c tablet
1140868364	prednisone	1140874940	deltastab 1mg tablet
1140868370	decortisyl 5mg tablet	1140874944	precortisyl 1mg tablet
1140868426	triamcinolone	1140874950	prednesol 5mg tablet
1140868434	ledercort 2mg tablet	1140874954	hydrocortistab 20mg tablet
1140874790	betamethasone	1140874956	hydrocortone 10mg tablet
1140874792	betnelan 500mcg tablet	1140874976	methylprednisolone
1140874794	betnesol 500mcg soluble tablet	1140874978	medrone 2mg tablet
1140874810	cortistab 5mg tablet	1140884704	cortisone product
1140874814	cortisyl 25mg tablet	1141157402	prednisolone product
1140874816	dexamethasone	1141173346	cortisone
1140874822	decadron 500micrograms tablet		

HRT – hormone replacement therapy; **OC** – oral contraceptive. Coding 4 in UK Biobank.

Supplementary Table S2 Characteristics of study participants

	MEN			WOMEN				
	Overall	< 55 years	≥ 55 years	Overall	Pre-MP	Post-MP Never-HRT	Post-MP Past-HRT	Post-MP Current-HRT
Cohort size: n (% per sex)	179,902	69,378 (38.6)	110,524 (61.4)	207,444	40,956 (19.7)	63,134 (30.4)	54,591 (26.3)	10,722 (5.2)
Anthropometry: mean (SD)								
Height (cm)	175.9 (6.8)	177.2 (6.8)	175.1 (6.6)	162.7 (6.2)	164.4 (6.2)	162.2 (6.2)	161.7 (6.1)	162.3 (6.0)
Weight (kg)	86 (13.7)	87.0 (14.2)	85.4 (13.4)	71.1 (13.1)	70.6 (13.4)	70.9 (13.0)	71.3 (12.6)	69.2 (11.8)
Weight change, last year: n (%)								
Lost weight	25,980 (14.4)	10,581 (15.3)	15,399 (13.9)	31,425 (15.1)	6,269 (15.3)	9,012 (14.3)	8,414 (15.4)	1,488 (13.9)
Stable weight	110,892 (61.6)	39,989 (57.6)	70,903 (64.2)	105,860 (51.0)	20,447 (49.9)	34,420 (54.5)	27,964 (51.2)	5,664 (52.8)
Gained weight	40,003 (22.2)	17,432 (25.1)	22,571 (20.4)	66,943 (32.3)	13,556 (33.1)	18,596 (29.5)	17,519 (32.1)	3,463 (32.3)
Missing	3,027 (1.7)	1,376 (2.0)	1,651 (1.5)	3,216 (1.6)	684 (1.7)	1,106 (1.8)	694 (1.3)	107 (1.0)
Smoking status: n (%)								
Never smoked	61,999 (34.5)	27,638 (39.8)	34,361 (31.1)	90,754 (43.7)	18,898 (46.1)	29,129 (46.1)	21,703 (39.8)	4,247 (39.6)
Former occasional smoker	45,779 (25.4)	18,717 (27.0)	27,062 (24.5)	56,936 (27.4)	11,877 (29.0)	17,436 (27.6)	14,605 (26.8)	2,911 (27.1)
Former regular smoker	49,675 (27.6)	12,473 (18.0)	37,202 (33.7)	41,003 (19.8)	5,975 (14.6)	11,934 (18.9)	13,608 (24.9)	2,584 (24.1)
Current smoker	21,877 (12.2)	10,418 (15.0)	11,459 (10.4)	18,099 (8.7)	4,136 (10.1)	4,436 (7.0)	4,457 (8.2)	950 (8.9)
Missing	572 (0.3)	132 (0.2)	440 (0.4)	652 (0.3)	70 (0.2)	199 (0.3)	218 (0.4)	30 (0.3)
Alcohol intake: n (%)								
Up to three times a month	36,257 (20.2)	14,811 (21.3)	21,446 (19.4)	72,189 (34.8)	12,779 (31.2)	22,745 (36.0)	19,283 (35.3)	3,342 (31.2)
Up to four times a week	96,455 (53.6)	39,976 (57.6)	56,479 (51.1)	100,164 (48.3)	22,205 (54.2)	29,760 (47.1)	24,996 (45.8)	5,013 (46.8)
Daily	47,047 (26.2)	14,522 (20.9)	32,525 (29.4)	34,975 (16.9)	5,959 (14.5)	10,595 (16.8)	10,277 (18.8)	2,360 (22.0)
Missing	143 (0.1)	69 (0.1)	74 (0.1)	116 (0.1)	13 (0.0)	34 (0.1)	35 (0.1)	7 (0.1)
Physical activity: n (%)								
Inactive	26,919 (15.0)	9,800 (14.1)	17,119 (15.5)	34,261 (16.5)	6,616 (16.2)	10,202 (16.2)	8,913 (16.3)	1,699 (15.8)
Moderately active	80,281 (44.6)	26,901 (38.8)	53,380 (48.3)	108,067 (52.1)	19,327 (47.2)	34,360 (54.4)	29,967 (54.9)	5,599 (52.2)
Active	72,145 (40.1)	32,487 (46.8)	39,658 (35.9)	64,420 (31.1)	14,955 (36.5)	18,351 (29.1)	15,508 (28.4)	3,390 (31.6)
Missing	557 (0.3)	190 (0.3)	367 (0.3)	696 (0.3)	58 (0.1)	221 (0.4)	203 (0.4)	34 (0.3)
Townsend index								
Mean (SD)	-1.44 (3.03)	-1.17 (3.14)	-1.62 (2.95)	-1.52 (2.92)	-1.37 (2.98)	-1.63 (2.86)	-1.61 (2.88)	-1.58 (2.93)
Missing: n (%)	218 (0.1)	116 (0.2)	102 (0.1)	229 (0.1)	55 (0.1)	60 (0.1)	58 (0.1)	8 (0.1)
Time of sample collection: n (%)								
≥8 am to <12 am	48,127 (26.8)	20,502 (29.6)	27,625 (25.0)	51,106 (24.6)	11,988 (29.3)	14,805 (23.5)	12,035 (22.0)	2,461 (23.0)
≥12 am to <4 pm	69,220 (38.5)	22,827 (32.9)	46,393 (42.0)	88,973 (42.9)	15,185 (37.1)	27,758 (44.0)	26,055 (47.7)	4,652 (43.4)
≥4 pm	62,403 (34.7)	26,000 (37.5)	36,403 (32.9)	67,165 (32.4)	13,739 (33.5)	20,508 (32.5)	16,450 (30.1)	3,603 (33.6)
Missing	152 (0.1)	49 (0.1)	103 (0.1)	200 (0.1)	44 (0.1)	63 (0.1)	51 (0.1)	6 (0.1)
Fasting time: n (%)								
0-2 hours	47,354 (26.3)	21,323 (30.7)	26,031 (23.6)	54,371 (26.2)	12,962 (31.6)	15,508 (24.6)	12,893 (23.6)	2,610 (24.3)
3-4 hours	89,300 (49.6)	31,175 (44.9)	58,125 (52.6)	108,861 (52.5)	19,849 (48.5)	33,922 (53.7)	29,988 (54.9)	5,734 (53.5)
≥5 hours	43,246 (24.0)	16,879 (24.3)	26,367 (23.9)	44,206 (21.3)	8,143 (19.9)	13,703 (21.7)	11,709 (21.4)	2,378 (22.2)
Missing	2	1	1	6	2	1	1	-
Cholesterol lowering drugs: n (%)								
No	139,199 (77.4)	62,737 (90.4)	76,462 (69.2)	181,721 (87.6)	40,806 (99.6)	54,360 (86.1)	44,012 (80.6)	9,357 (87.3)
Yes	39,204 (21.8)	5,962 (8.6)	33,242 (30.1)	24,995 (12.0)	-	8,573 (13.6)	10,433 (19.1)	1,319 (12.3)
Missing	1,499 (0.8)	679 (1.0)	820 (0.7)	728 (0.4)	150 (0.4)	201 (0.3)	146 (0.3)	46 (0.4)

	MEN			WOMEN				
	Overall	< 55 years	≥ 55 years	Overall	Pre-MP	Post-MP Never-HRT	Post-MP Past-HRT	Post-MP Current-HRT
Assessment region: n (%)								
London	20,380 (11.3)	8,010 (11.5)	12,370 (11.2)	24,513 (11.8)	5,342 (13.0)	7,989 (12.7)	5,741 (10.5)	1,565 (14.6)
North-West	28,189 (15.7)	10,551 (15.2)	17,638 (16.0)	31,145 (15.0)	5,683 (13.9)	8,943 (14.2)	8,700 (15.9)	1,835 (17.1)
North-East	22,078 (12.3)	8,370 (12.1)	13,708 (12.4)	25,412 (12.3)	4,722 (11.5)	7,973 (12.6)	7,049 (12.9)	1,185 (11.1)
Yorkshire and Humber	27,352 (15.2)	10,231 (14.7)	17,121 (15.5)	31,612 (15.2)	5,916 (14.4)	9,879 (15.6)	8,293 (15.2)	1,297 (12.1)
West Midlands	16,717 (9.3)	6,315 (9.1)	10,402 (9.4)	17,068 (8.2)	3,229 (7.9)	5,087 (8.1)	4,734 (8.7)	929 (8.7)
East Midlands	12,467 (6.9)	4,394 (6.3)	8,073 (7.3)	14,386 (6.9)	2,688 (6.6)	4,437 (7.0)	4,064 (7.4)	670 (6.2)
South-East	15,837 (8.8)	6,284 (9.1)	9,553 (8.6)	19,083 (9.2)	3,783 (9.2)	5,520 (8.7)	4,898 (9.0)	1,159 (10.8)
South-West	15,729 (8.7)	6,557 (9.5)	9,172 (8.3)	19,053 (9.2)	4,208 (10.3)	5,633 (8.9)	4,464 (8.2)	986 (9.2)
Wales	7,873 (4.4)	3,151 (4.5)	4,722 (4.3)	9,018 (4.3)	1,894 (4.6)	2,657 (4.2)	2,370 (4.3)	383 (3.6)
Scotland	13,280 (7.4)	5,515 (7.9)	7,765 (7.0)	16,154 (7.8)	3,491 (8.5)	5,016 (7.9)	4,278 (7.8)	713 (6.6)
OC use with time stopped: n (%)								
Never	-	-	-	35,855 (17.3)	4,077 (10.0)	15,171 (24.0)	10,728 (19.7)	1,552 (14.5)
Past <10 (or <20) years	-	-	-	29,976 (14.5)	8,063 (19.7)	7,906 (12.5)	5,033 (9.2)	1,829 (17.1)
Past ≥10 to <20 (or ≥20 to <30)	-	-	-	58,269 (28.1)	15,400 (37.6)	16,991 (26.9)	13,788 (25.3)	3,218 (30.0)
Past ≥20 (≥30) years	-	-	-	58,124 (28.0)	11,359 (27.7)	18,452 (29.2)	18,610 (34.1)	3,119 (29.1)
Past Unknown time	-	-	-	16,632 (8.0)	2,057 (5.0)	4,614 (7.3)	6,432 (11.8)	1,004 (9.4)
Current	-	-	-	8,588 (4.1)	-	-	-	-
Age at the last live birth: n (%)								
No live births	-	-	-	38,133 (18.4)	10,320 (25.2)	10,627 (16.8)	7,480 (13.7)	2,050 (19.1)
< 30 years	-	-	-	80,289 (38.7)	9,923 (24.2)	24,220 (38.4)	26,057 (47.7)	4,588 (42.8)
≥ 30 years	-	-	-	88,580 (42.7)	20,664 (50.5)	28,154 (44.6)	20,925 (38.3)	4,066 (37.9)
Missing	-	-	-	442 (0.2)	49 (0.1)	133 (0.2)	129 (0.2)	18 (0.2)
Age at menarche								
Mean (SD)	-	-	-	13 (1.6)	13.1 (1.6)	13.0 (1.6)	12.9 (1.6)	13.0 (1.7)
Missing: n (%)	-	-	-	5616 (2.7)	1151 (2.8)	1799 (2.8)	1329 (2.4)	255 (2.4)
Age at menopause								
Mean (SD)	-	-	-	49.5 (5.3)	-	50.6 (4.2)	49.1 (5.7)	48.1 (6.1)
Missing: n (%)	-	-	-	80,946 (39)	-	2,350 (3.7)	4,472 (8.2)	807 (7.5)
Oophorectomy (bilateral)								
Yes: n (%)	-	-	-	14,568 (7.0)	-	1,798 (2.8)	8,423 (15.4)	3,061 (28.5)
Missing values (Table 1)								
Missing hand grip strength: n (%)	162 (0.1)	53 (0.1)	109 (0.1)	233 (0.1)	31 (0.1)	82 (0.1)	73 (0.1)	9 (0.1)

OC – oral contraceptives, cut-offs for duration of use correspond to pre-menopausal (post-menopausal or undetermined) menopausal status; **SD** – standard deviation; **n (%)** – number (percentage from total per column); # – number (percentage from Post-MP Current-HRT, overall).

The following subgroup-specific variables were additionally used as covariates:

Women (overall): Menopausal status: Pre-MP (n=50,107, 24.2%), Post-MP (n=134,419, 64.8%), Undetermined (n=22,918, 11.0%); Hormone replacement therapy (HRT) use and duration: Never use (n=126,558, 61.0%), Past use <6 years (n=28,019, 13.5%), Past use ≥6 years (n=25,888,

12.5%), Past use Unknown duration (n=9,067, 4.4%), Current use <11 years (n=9,080, 4.4%), Current use ≥11 years (n=7,179, 3.5%), Current use Unknown duration (n=1,653, 0.8%).

Pre-MP: Time of the menstrual period: Early follicular (days 0-5, n=10,064, 24.6%), Late follicular (days 6-10, n=7,122, 17.4%); Mid-cycle (days 11-14, n=5,378, 13.1%); Early luteal (days 15-18, n=3,557, 8.7%); Mid-luteal (days 19-24, n=6,042, 14.8%); Late luteal (days 25-40, n=3,924, 9.6%); Undetermined (days ≥41 or missing, n=4,869, 11.9%).

Post-MP Past-HRT: Duration of HRT use: <6 years (n=24,290, 44.5%), ≥6 years (n=22,668, 41.5%), Unknown duration (n=7,633, 14.0%); Time since stopped HRT: <7 years (n=24,857, 45.5%), ≥7 years (n=23,446, 42.9%), Unknown (n=6,288, 11.5%).

Post-MP Current-HRT: Duration of HRT use: <11 years (n=4,764, 44.4%), ≥11 years (n=4,890, 45.6%), Unknown (n=1,068, 10.0%); Type of HRT: Combined or Progestin-only (n=3,632, 33.9%), Oestrogen-only (n=5,238, 48.9%), Unknown (n=1,852, 17.3%).

Supplementary Table S3 Associations of sex steroids and their binding proteins with age at enrolment

	Men	Women Pre-MP	Women Post-MP Never-HRT	Women Post-MP Past-HRT	Women Post-MP Current-HRT
**Age at enrolment: (per 5 years)**					
SHBG: SD_{diff} (95% CI)	0.21 (0.21 to 0.22)**	0.07 (0.05 to 0.08)**	0.06 (0.06 to 0.07)**	0.10 (0.09 to 0.11)**	-0.01 (-0.04 to 0.02)
Albumin: SD_{diff} (95% CI)	-0.17 (-0.18 to -0.17)**	-0.15 (-0.16 to -0.13)**	-0.16 (-0.16 to -0.15)**	-0.15 (-0.16 to -0.14)**	-0.12 (-0.14 to -0.09)**
T: SD_{diff} (95% CI)	-0.01 (-0.01 to 0.00)**	-0.12 (-0.13 to -0.11)**	-0.05 (-0.05 to -0.04)**	-0.05 (-0.06 to -0.04)**	-0.13 (-0.16 to -0.11)**
fT: SD_{diff} (95% CI)	-0.12 (-0.13 to -0.12)**	-0.13 (-0.14 to -0.12)**	-0.05 (-0.06 to -0.04)**	-0.07 (-0.08 to -0.06)**	-0.12 (-0.15 to -0.09)**
E2: OR (95% CI)	1.00 (0.99 to 1.02)	0.64 (0.61 to 0.66)**	0.39 (0.37 to 0.42)**	0.67 (0.62 to 0.73)**	0.72 (0.69 to 0.77)**
E2: SD_{diff} (95% CI)	-	-0.11 (-0.12 to -0.09)**	-	-	-
fE2: SD_{diff} (95% CI)	-	-0.12 (-0.13 to -0.10)**	-	-	-

E2 – oestradiol; **fE2** – free oestradiol; **fT** – free testosterone; **HRT** – hormone replacement therapy; **Post-MP** – post-menopausal; **Pre-MP** – pre-menopausal; **SHBG** – sex hormone binding globulin; **T** – testosterone. **OR (95% CI)** – estimates for odds ratios of oestradiol detection (95% confidence interval) were obtained from multivariable logistic regression models. **SD_{diff} (95% CI)** – estimates for standard deviation differences (95% confidence interval) were obtained from linear regression models including as an outcome variable SHBG, albumin, T, or fT on a continuous standard deviation scale (sex-specific z-scores). All models included as independent variables a body shape index (ABSI), hip index (HI), body mass index (BMI) and height (sex-specific z-scores, continuous scale), age at enrolment, weight change during the last year preceding enrolment, smoking status, alcohol consumption, physical activity, Townsend deprivation index, region of the assessment centre, time of sample collection, fasting time, use of cholesterol lowering drugs (except Pre-MP) and in women age at the last live birth, oral contraceptive use with time since stopped, bilateral oophorectomy (except Pre-MP) and, additionally, time of the menstrual period (Pre-MP), duration of use and time since stopped HRT (Past-HRT), duration of use and type of HRT (Current-HRT). Participant groups are defined in Supplementary Figure S1. * – p<0.05 from Wald test for the individual term; ** – p<0.0001.

Supplementary Table S4 Associations of sex steroids and their binding proteins with body size and body shape indices (continuous)

Group	Count	BMI	ABSI	HI
**SHBG** SD_{diff} (95% CI)				
Men (overall)	165,290	-0.29 (-0.29 to -0.28)**	-0.07 (-0.08 to -0.07)**	0.08 (0.08 to 0.09)**
Men <55 years	63,753	-0.32 (-0.33 to -0.31)**	-0.10 (-0.11 to -0.09)**	0.10 (0.09 to 0.11)**
Men ≥55 years	101,537	-0.26 (-0.27 to -0.26)**	-0.06 (-0.07 to -0.05)**	0.08 (0.07 to 0.08)**
Women (overall)	187,462	-0.41 (-0.41 to -0.40)**	-0.16 (-0.17 to -0.16)**	0.12 (0.11 to 0.12)**
Pre-MP	36,848	-0.41 (-0.42 to -0.40)**	-0.15 (-0.15 to -0.14)**	0.09 (0.08 to 0.10)**
Post-MP Never-HRT	57,185	-0.41 (-0.42 to -0.40)**	-0.18 (-0.19 to -0.17)**	0.13 (0.12 to 0.14)**
Post-MP Past-HRT	49,447	-0.40 (-0.41 to -0.39)**	-0.17 (-0.18 to -0.16)**	0.11 (0.11 to 0.12)**
Post-MP Current-HRT	9,655	-0.36 (-0.39 to -0.33)**	-0.16 (-0.18 to -0.13)**	0.13 (0.11 to 0.16)**
**Albumin** SD_{diff} (95% CI)				
Men (overall)	165,869	-0.08 (-0.08 to -0.07)**	-0.03 (-0.03 to -0.02)**	-0.02 (-0.03 to -0.02)**
Men <55 years	63,971	-0.10 (-0.10 to -0.09)**	-0.03 (-0.03 to -0.02)**	-0.01 (-0.02 to 0.00)*
Men ≥55 years	101,898	-0.06 (-0.07 to -0.06)**	-0.03 (-0.03 to -0.02)**	-0.03 (-0.04 to -0.02)**
Women (overall)	188,202	-0.19 (-0.19 to -0.18)**	0.00 (0.00 to 0.01)	-0.05 (-0.06 to -0.05)**
Pre-MP	36,958	-0.23 (-0.24 to -0.22)**	0.00 (-0.01 to 0.01)	-0.04 (-0.05 to -0.03)**
Post-MP Never-HRT	57,404	-0.18 (-0.19 to -0.17)**	0.00 (-0.01 to 0.01)	-0.06 (-0.07 to -0.05)**
Post-MP Past-HRT	49,625	-0.17 (-0.18 to -0.16)**	0.00 (-0.01 to 0.01)	-0.06 (-0.07 to -0.05)**
Post-MP Current-HRT	9,731	-0.15 (-0.18 to -0.13)**	0.01 (-0.01 to 0.03)	-0.05 (-0.07 to -0.03)**
**Total Testosterone SDdiff (95% CI)**				
Men (overall)	179,033	-0.28 (-0.28 to -0.27)**	-0.08 (-0.08 to -0.07)**	0.01 (0.01 to 0.02)**
Men <55 years	69,042	-0.27 (-0.28 to -0.26)**	-0.08 (-0.09 to -0.07)**	0.02 (0.01 to 0.03)**
Men ≥55 years	109,991	-0.28 (-0.29 to -0.27)**	-0.08 (-0.08 to -0.07)**	0.01 (0.01 to 0.02)**
Women (overall)	206,611	0.11 (0.10 to 0.11)**	-0.02 (-0.03 to -0.02)**	0.00 (0.00 to 0.01)*
Pre-MP	40,824	0.08 (0.07 to 0.08)**	0.00 (-0.01 to 0.00)	0.00 (-0.01 to 0.01)
Post-MP Never-HRT	62,864	0.12 (0.11 to 0.13)**	-0.03 (-0.04 to -0.02)**	0.01 (0.00 to 0.01)
Post-MP Past-HRT	54,358	0.12 (0.11 to 0.13)**	-0.02 (-0.03 to -0.02)**	0.00 (-0.01 to 0.01)
Post-MP Current-HRT	10,681	0.08 (0.06 to 0.11)**	-0.02 (-0.05 to 0.00)*	0.01 (-0.01 to 0.03)
**Free Testosterone** SD_{diff} (95% CI)				
Men (overall)	164,607	-0.14 (-0.14 to -0.13)**	-0.04 (-0.05 to -0.04)**	-0.04 (-0.04 to -0.03)**
Men <55 years	63,497	-0.12 (-0.13 to -0.11)**	-0.04 (-0.04 to -0.03)**	-0.03 (-0.04 to -0.03)**
Men ≥55 years	101,110	-0.15 (-0.16 to -0.14)**	-0.05 (-0.05 to -0.04)**	-0.04 (-0.04 to -0.03)**
Women (overall)	186,764	0.22 (0.21 to 0.22)**	0.02 (0.02 to 0.03)**	-0.03 (-0.03 to -0.02)**
Pre-MP	36,720	0.20 (0.19 to 0.20)**	0.04 (0.03 to 0.04)**	-0.02 (-0.03 to -0.02)**
Post-MP Never-HRT	56,976	0.23 (0.22 to 0.24)**	0.02 (0.01 to 0.03)**	-0.03 (-0.04 to -0.02)**
Post-MP Past-HRT	49,253	0.23 (0.22 to 0.24)**	0.02 (0.01 to 0.03)**	-0.03 (-0.04 to -0.02)**
Post-MP Current-HRT	9,622	0.19 (0.16 to 0.22)**	0.02 (0.00 to 0.04)	-0.03 (-0.05 to 0.00)*
**Total Oestradiol** OR (95% CI)				
Men (overall)	168,273	1.14 (1.12 to 1.16)**	0.98 (0.96 to 1.00)*	1.04 (1.02 to 1.05)**
Men <55 years	64,835	1.16 (1.13 to 1.19)**	0.98 (0.96 to 1.01)	1.02 (1.00 to 1.05)
Men ≥55 years	103,438	1.13 (1.11 to 1.16)**	0.97 (0.95 to 1.00)*	1.04 (1.02 to 1.07)**
Women (overall)	194,092	1.05 (1.03 to 1.07)**	0.93 (0.92 to 0.95)**	1.01 (1.00 to 1.03)
Pre-MP	38,324	0.95 (0.93 to 0.98)*	0.93 (0.90 to 0.95)**	1.03 (1.00 to 1.06)*
Post-MP Never-HRT	59,087	1.25 (1.19 to 1.31)**	0.96 (0.92 to 1.01)	1.03 (0.99 to 1.08)
Post-MP Past-HRT	51,103	1.33 (1.25 to 1.42)**	0.98 (0.92 to 1.05)	1.02 (0.96 to 1.08)
Post-MP Current-HRT	10,027	0.99 (0.94 to 1.04)	0.96 (0.92 to 1.01)	1.04 (0.99 to 1.08)
**Total Oestradiol** SD_{diff} (95% CI)				
Pre-MP	38,324	-0.04 (-0.05 to -0.03)**	-0.04 (-0.05 to -0.03)**	0.01 (0.00 to 0.02)*
**Free Oestradiol** SD_{diff} (95% CI)				
Pre-MP	34,280	0.05 (0.04 to 0.06)**	-0.02 (-0.03 to -0.01)*	0.00 (-0.01 to 0.01)

ABSI – a body shape index; **BMI** – body mass index; **Count** – number of participants with available biomarker measurements included in the model; **HI** – hip index; **HRT** – hormone replacement therapy; **Post-MP** – post-menopausal; **Pre-MP** – pre-menopausal; **SHBG** – sex hormone binding globulin.

SD_{diff} (95% CI) – estimates for standard deviation differences (95% confidence interval) were obtained from multivariable linear regression models with SHBG, albumin, total testosterone, free testosterone, or total or free oestradiol (Pre-MP) as an outcome variable; **OR (95% CI)** – estimates for odds ratios of oestradiol detection (95% confidence interval) were obtained from multivariable logistic regression models. All models included ABSI, HI and BMI on a continuous standard deviation scale (sex-specific z-scores), with adjustment for height, age at enrolment, weight change within the last year preceding enrolment, smoking status, alcohol consumption, physical activity, Townsend deprivation index, region of the assessment centre, time of blood collection, fasting time, use of cholesterol lowering drugs (except Pre-MP), and in women also age at the last live birth, oral contraceptives use with time since stopped, bilateral oophorectomy (except Pre-MP) and, additionally, menopausal status and HRT use and duration (women overall), time of the menstrual period (Pre-MP), time since stopped and duration of HRT use (Past-HRT), or duration of use and type of HRT (Current-HRT). Participant groups are defined in Supplementary Figure S1. Covariates are defined in Supplementary Methods.

* – $p<0.05$ from Wald test for the individual term; ** – $p<0.0001$.

Supplementary Table S5 Associations of sex steroids and their binding proteins with body mass index (categorical)

**Group**		**SHBG**		**Albumin**		**Free Testosterone**		**Total Testosterone**		**Total Oestradiol**
	Count	SD_{diff} (95% CI)	Count	SD_{diff} (95% CI)	Count	SD_{diff} (95% CI)	Count	SD_{diff} (95% CI)	Count	OR (95% CI)
**Men (overall)**										
NW	41,169	reference	41,320	reference	44,785	reference	40,993	reference	42,118	reference
OW	82,472	-0.48 (-0.49 to -0.47)**	82,718	-0.01 (-0.02 to 0.00)	89,360	-0.05 (-0.06 to -0.04)**	82,160	-0.31 (-0.32 to -0.30)**	83,981	1.06 (1.01 to 1.10)*
OB	41,649	-0.77 (-0.78 to -0.75)**	41,831	-0.15 (-0.16 to -0.13)**	44,888	-0.31 (-0.32 to -0.30)**	41,454	-0.68 (-0.70 to -0.67)**	42,174	1.29 (1.23 to 1.36)**
**Men (<55 years)**										
NW	17,034	reference	17,099	reference	18,518	reference	16,960	reference	17,416	reference
OW	31,002	-0.55 (-0.57 to -0.53)**	31,107	-0.04 (-0.06 to -0.03)**	33,659	-0.04 (-0.06 to -0.03)**	30,898	-0.33 (-0.34 to -0.31)**	31,629	1.08 (1.01 to 1.16)*
OB	15,717	-0.88 (-0.90 to -0.86)**	15,765	-0.20 (-0.22 to -0.18)**	16,865	-0.28 (-0.30 to -0.25)**	15,639	-0.69 (-0.71 to -0.67)**	15,790	1.33 (1.23 to 1.43)**
**Men (≥55 years)**										
NW	24,135	reference	24,221	reference	26,267	reference	24,033	reference	24,702	reference
OW	51,470	-0.43 (-0.44 to -0.41)**	51,611	0.01 (0.00 to 0.03)	55,701	-0.06 (-0.08 to -0.05)**	51,262	-0.30 (-0.32 to -0.29)**	52,352	1.04 (0.98 to 1.10)
OB	25,932	-0.69 (-0.71 to -0.67)**	26,066	-0.11 (-0.13 to -0.09)**	28,023	-0.33 (-0.35 to -0.32)**	25,815	-0.68 (-0.70 to -0.66)**	26,384	1.27 (1.19 to 1.35)**
**Women (overall)**										
NW	75,438	reference	75,731	reference	83,395	reference	75,161	reference	78,398	reference
OW	69,789	-0.47 (-0.47 to -0.46)**	70,059	-0.17 (-0.18 to -0.16)**	76,933	0.27 (0.26 to 0.28)**	69,543	0.13 (0.12 to 0.14)**	72,251	1.02 (0.98 to 1.05)
OB	42,235	-0.97 (-0.98 to -0.96)**	42,412	-0.43 (-0.45 to -0.42)**	46,283	0.52 (0.50 to 0.53)**	42,060	0.25 (0.23 to 0.26)**	43,443	1.11 (1.06 to 1.15)**
**Women Pre-MP**										
NW	18,162	reference	18,214	reference	20,169	reference	18,096	reference	18,960	reference
OW	11,911	-0.44 (-0.46 to -0.42)**	11,951	-0.22 (-0.24 to -0.19)**	13,180	0.25 (0.23 to 0.27)**	11,870	0.12 (0.10 to 0.13)**	12,376	0.98 (0.93 to 1.04)
OB	6,775	-1.02 (-1.04 to -0.99)**	6,793	-0.55 (-0.57 to -0.52)**	7,475	0.47 (0.45 to 0.49)**	6,754	0.17 (0.15 to 0.19)**	6,988	0.87 (0.81 to 0.93)*
**Women Post-MP Never-HRT**										
NW	22,551	reference	22,629	reference	24,888	reference	22,464	reference	23,397	reference
OW	21,575	-0.50 (-0.51 to -0.48)**	21,660	-0.15 (-0.17 to -0.13)**	23,730	0.27 (0.26 to 0.29)**	21,503	0.14 (0.12 to 0.16)**	22,284	1.15 (1.03 to 1.28)*
OB	13,059	-0.98 (-1.00 to -0.96)**	13,115	-0.43 (-0.45 to -0.41)**	14,246	0.54 (0.51 to 0.56)**	13,009	0.27 (0.25 to 0.29)**	13,406	1.67 (1.47 to 1.88)**
**Women Post-MP Past-HRT**										
NW	17,410	reference	17,465	reference	19,174	reference	17,348	reference	18,033	reference
OW	20,109	-0.46 (-0.48 to -0.44)**	20,182	-0.14 (-0.16 to -0.12)**	22,110	0.28 (0.26 to 0.30)**	20,030	0.15 (0.13 to 0.17)**	20,782	1.23 (1.04 to 1.46)*
OB	11,928	-0.94 (-0.96 to -0.92)**	11,978	-0.37 (-0.39 to -0.34)**	13,074	0.53 (0.50 to 0.56)**	11,875	0.28 (0.25 to 0.30)**	12,288	2.02 (1.70 to 2.41)**
**Women Post-MP Current-HRT**										
NW	4,303	reference	4,340	reference	4,750	reference	4,292	reference	4,458	reference
OW	3,650	-0.40 (-0.46 to -0.35)**	3,677	-0.15 (-0.19 to -0.10)**	4,076	0.24 (0.18 to 0.29)**	3,637	0.11 (0.06 to 0.16)**	3,809	0.99 (0.90 to 1.10)
OB	1,702	-0.78 (-0.86 to -0.71)**	1,714	-0.33 (-0.39 to -0.27)**	1,855	0.42 (0.35 to 0.49)**	1,693	0.18 (0.12 to 0.25)**	1,760	0.99 (0.87 to 1.13)
**Group**								**Free Oestradiol**		**Total Oestradiol**
							Count	SD_{diff} (95% CI)	Count	SD_{diff} (95% CI)
**Women Pre-MP** (oestradiol on a continuous scale, with imputed undetected values)										
NW							16,916	reference	18,960	reference
OW							11,087	0.07 (0.05 to 0.10)**	12,376	-0.03 (-0.05 to -0.01)*
OB							6,277	0.11 (0.08 to 0.14)**	6,988	-0.11 (-0.14 to -0.08)**

BMI – body mass index; **Count** – number of participants with available biomarker measurements per category; **HRT** – hormone replacement therapy; **NW** – normal weight (BMI≥18.5 to <25 kg/m^2); **OB** – obese (BMI≥30 to <45 kg/m^2); **OR** – odds ratio; **OW** – overweight (BMI≥25 to <30 kg/m^2); **Post-MP** – post-menopausal; **Pre-MP** – pre-menopausal; **SHBG** – sex hormone binding globulin.

SD_{diff} (95% CI) – estimates for standard deviation differences (95% confidence interval) were obtained from multivariable linear regression models including SHBG, albumin, total testosterone, free testosterone, or total or free oestradiol (Pre-MP) on a continuous standard deviation scale (sex-specific z-scores) as an outcome variable and as independent variables BMI categories; **OR (95% CI)** – estimates for odds ratios of oestradiol detection (95% confidence interval) were obtained from multivariable logistic regression models. All models were adjusted for an ABSI-by-HI cross-classification variable (a body shape index with cut-offs ≥80 for men, ≥73 for women, hip index with cut-offs ≥49 for men, ≥64 for women), height, age at enrolment, weight change within the last year preceding enrolment, smoking status, alcohol consumption, physical activity, Townsend deprivation index, region of the assessment centre, time of blood collection, fasting time, use of cholesterol lowering drugs (except Pre-MP), and in women also age at the last live birth, oral contraceptives use with time since stopped, bilateral oophorectomy (except Pre-MP) and, additionally, menopausal status and HRT use and duration (women overall), time of the menstrual period (Pre-MP), time since stopped and duration of HRT use (Past-HRT), or duration of use and type of HRT (Current-HRT). Participant groups are defined in Supplementary Figure S1. Covariates are defined in Supplementary Methods.

* – p<0.05 from Wald test for the individual term; ** – p<0.0001.

Supplementary Table S6 Associations of sex steroids and their binding proteins with body shape phenotypes (categorical)

Group		SHBG Count	SHBG SD_{diff} (95% CI)	Albumin Count	Albumin SD_{diff} (95% CI)	Testosterone Count	Testosterone SD_{diff} (95% CI)	Free Testosterone Count	Free Testosterone SD_{diff} (95% CI)	Oestradiol (binary) Count	Oestradiol (binary) OR (95% CI)
**Men (overall)**											
ALL	Pear	39,655	reference	39,801	reference	39,496	reference	42,984	reference	40,374	reference
	Slim	47,341	-0.11 (-0.13 to -0.10)**	47,521	0.04 (0.02 to 0.05)**	47,172	0.05 (0.04 to 0.07)**	51,315	-0.01 (-0.02 to 0.00)	48,209	0.97 (0.93 to 1.02)
	Wide	47,780	-0.10 (-0.11 to -0.09)**	47,930	-0.04 (-0.06 to -0.03)**	47,557	-0.08 (-0.10 to -0.07)**	51,723	-0.13 (-0.14 to -0.11)**	48,695	1.00 (0.95 to 1.04)
	Apple	30,514	-0.20 (-0.22 to -0.19)**	30,617	-0.02 (-0.03 to 0.00)*	30,382	-0.01 (-0.03 to 0.00)	33,011	-0.12 (-0.14 to -0.11)**	30,995	0.94 (0.89 to 0.99)*
	p-value		$1*10^{-190}$		$4*10^{-34}$		$2*10^{-103}$		$1*10^{-142}$		0.068
NW	Pear	11,891	reference	11,931	reference	11,841	reference	12,931	reference	12,204	reference
	Slim	11,067	-0.11 (-0.13 to -0.09)**	11,104	0.00 (-0.02 to 0.03)	11,025	0.04 (0.02 to 0.07)*	12,017	-0.03 (-0.05 to -0.01)*	11,300	0.99 (0.90 to 1.08)
	Wide	12,354	-0.18 (-0.20 to -0.16)**	12,403	-0.03 (-0.06 to -0.01)*	12,298	-0.05 (-0.07 to -0.02)*	13,469	-0.14 (-0.16 to -0.12)**	12,664	1.00 (0.91 to 1.09)
	Apple	5,857	-0.29 (-0.32 to -0.26)**	5,882	-0.03 (-0.06 to 0.00)*	5,829	0.03 (0.01 to 0.06)*	6,368	-0.15 (-0.17 to -0.12)**	5,950	0.92 (0.82 to 1.04)
OW	Pear	19,018	-0.51 (-0.53 to -0.49)**	19,083	-0.02 (-0.04 to 0.00)*	18,950	-0.03 (-0.05 to -0.01)*	20,589	-0.32 (-0.34 to -0.30)**	19,305	1.05 (0.97 to 1.14)
	Slim	25,023	-0.63 (-0.65 to -0.61)**	25,107	0.01 (-0.01 to 0.03)	24,938	0.01 (-0.01 to 0.03)	27,143	-0.34 (-0.36 to -0.32)**	25,484	1.05 (0.97 to 1.14)
	Wide	22,640	-0.60 (-0.62 to -0.58)**	22,692	-0.05 (-0.07 to -0.03)**	22,551	-0.11 (-0.13 to -0.09)**	24,526	-0.44 (-0.46 to -0.42)**	23,117	1.04 (0.96 to 1.12)
	Apple	15,791	-0.72 (-0.74 to -0.70)**	15,836	-0.04 (-0.06 to -0.01)*	15,721	-0.07 (-0.09 to -0.04)**	17,102	-0.46 (-0.48 to -0.44)**	16,075	0.99 (0.91 to 1.08)
OB	Pear	8,746	-0.85 (-0.88 to -0.83)**	8,787	-0.16 (-0.19 to -0.14)**	8,705	-0.29 (-0.32 to -0.27)**	9,464	-0.73 (-0.75 to -0.70)**	8,865	1.32 (1.20 to 1.45)**
	Slim	11,251	-0.94 (-0.96 to -0.92)**	11,310	-0.07 (-0.10 to -0.05)**	11,209	-0.21 (-0.23 to -0.18)**	12,155	-0.69 (-0.71 to -0.67)**	11,425	1.19 (1.08 to 1.30)*
	Wide	12,786	-0.88 (-0.90 to -0.85)**	12,835	-0.24 (-0.27 to -0.22)**	12,708	-0.42 (-0.44 to -0.39)**	13,728	-0.84 (-0.87 to -0.82)**	12,914	1.32 (1.21 to 1.45)**
	Apple	8,866	-0.96 (-0.99 to -0.94)**	8,899	-0.17 (-0.20 to -0.14)**	8,832	-0.30 (-0.33 to -0.27)**	9,541	-0.79 (-0.81 to -0.76)**	8,970	1.23 (1.12 to 1.36)**
	p-value		$1*10^{-32}$		$2*10^{-14}$		$4*10^{-13}$		$2*10^{-8}$		0.364
**Men (<55 years)**											
ALL	Pear	18,503	reference	18,570	reference	18,428	reference	20,079	reference	18,841	reference
	Slim	22,522	-0.12 (-0.14 to -0.10)**	22,604	0.02 (0.00 to 0.04)*	22,445	0.05 (0.03 to 0.07)**	24,401	-0.01 (-0.03 to 0.01)	22,894	0.98 (0.91 to 1.04)
	Wide	13,537	-0.12 (-0.14 to -0.10)**	13,575	-0.05 (-0.07 to -0.02)**	13,474	-0.08 (-0.10 to -0.06)**	14,644	-0.13 (-0.15 to -0.11)**	13,790	0.99 (0.91 to 1.07)
	Apple	9,191	-0.24 (-0.26 to -0.21)**	9,222	-0.05 (-0.07 to -0.02)*	9,150	0.00 (-0.03 to 0.02)	9,918	-0.12 (-0.14 to -0.10)**	9,310	0.99 (0.91 to 1.08)
	p-value		$2*10^{-90}$		$2*10^{-12}$		$4*10^{-34}$		$3*10^{-53}$		0.917
NW	Pear	5,588	reference	5,607	reference	5,561	reference	6,072	reference	5,719	reference
	Slim	5,505	-0.10 (-0.14 to -0.07)**	5,528	-0.02 (-0.05 to 0.02)	5,485	0.04 (0.00 to 0.07)*	5,964	-0.03 (-0.06 to 0.01)	5,589	0.98 (0.86 to 1.12)
	Wide	3,958	-0.17 (-0.21 to -0.13)**	3,972	-0.04 (-0.07 to 0.00)	3,938	-0.03 (-0.07 to 0.01)	4,310	-0.12 (-0.15 to -0.08)**	4,075	1.03 (0.89 to 1.19)
	Apple	1,983	-0.29 (-0.33 to -0.24)**	1,992	-0.07 (-0.12 to -0.02)*	1,976	0.04 (-0.01 to 0.09)	2,172	-0.13 (-0.18 to -0.09)**	2,033	0.90 (0.75 to 1.09)
OW	Pear	8,899	-0.55 (-0.58 to -0.52)**	8,935	-0.06 (-0.09 to -0.03)*	8,871	-0.02 (-0.05 to 0.01)	9,668	-0.32 (-0.35 to -0.29)**	9,065	1.06 (0.94 to 1.19)
	Slim	11,631	-0.68 (-0.71 to -0.65)**	11,673	-0.05 (-0.08 to -0.02)*	11,598	0.02 (-0.01 to 0.05)	12,641	-0.35 (-0.37 to -0.32)**	11,882	1.09 (0.97 to 1.22)
	Wide	6,052	-0.69 (-0.73 to -0.66)**	6,068	-0.08 (-0.11 to -0.04)**	6,031	-0.12 (-0.15 to -0.08)**	6,577	-0.47 (-0.50 to -0.44)**	6,195	1.01 (0.89 to 1.15)
	Apple	4,420	-0.81 (-0.85 to -0.77)**	4,431	-0.09 (-0.13 to -0.06)**	4,398	-0.04 (-0.07 to 0.00)*	4,773	-0.46 (-0.49 to -0.42)**	4,487	1.11 (0.97 to 1.28)
OB	Pear	4,016	-0.94 (-0.98 to -0.90)**	4,028	-0.23 (-0.27 to -0.19)**	3,996	-0.27 (-0.31 to -0.24)**	4,339	-0.73 (-0.77 to -0.69)**	4,057	1.39 (1.21 to 1.59)**
	Slim	5,386	-1.03 (-1.07 to -1.00)**	5,403	-0.14 (-0.17 to -0.10)**	5,362	-0.18 (-0.22 to -0.15)**	5,796	-0.68 (-0.72 to -0.65)**	5,423	1.21 (1.06 to 1.38)*
	Wide	3,527	-0.95 (-0.99 to -0.91)**	3,535	-0.33 (-0.37 to -0.29)**	3,505	-0.37 (-0.41 to -0.33)**	3,757	-0.82 (-0.85 to -0.78)**	3,520	1.37 (1.19 to 1.58)**
	Apple	2,788	-1.08 (-1.13 to -1.04)**	2,799	-0.27 (-0.31 to -0.22)**	2,776	-0.28 (-0.32 to -0.24)**	2,973	-0.80 (-0.84 to -0.76)**	2,790	1.32 (1.13 to 1.54)*
	p-value		$4*10^{-10}$		$5*10^{-10}$		$2*10^{-4}$		0.016		0.162

**Group**		Count	**SHBG** SD_{diff} (95% CI)	Count	**Albumin** SD_{diff} (95% CI)	Count	**Testosterone** SD_{diff} (95% CI)	Count	**Free Testosterone** SD_{diff} (95% CI)	Count	**Oestradiol** (binary) OR (95% CI)
**Men (≥55 years)**											
ALL	Pear	21,152	reference	21,231	reference	21,068	reference	22,905	reference	21,533	reference
	Slim	24,819	-0.11 (-0.12 to -0.09)**	24,917	0.05 (0.03 to 0.07)**	24,727	0.05 (0.04 to 0.07)**	26,914	-0.01 (-0.03 to 0.00)	25,315	0.96 (0.90 to 1.03)
	Wide	34,243	-0.09 (-0.10 to -0.07)**	34,355	-0.04 (-0.05 to -0.02)**	34,083	-0.09 (-0.10 to -0.07)**	37,079	-0.13 (-0.14 to -0.11)**	34,905	1.00 (0.94 to 1.06)
	Apple	21,323	-0.18 (-0.20 to -0.17)**	21,395	0.00 (-0.02 to 0.02)	21,232	-0.02 (-0.04 to 0.00)	23,093	-0.13 (-0.14 to -0.11)**	21,685	0.91 (0.85 to 0.98)*
	p-value		$2*10^{-100}$		$3*10^{-24}$		$5*10^{-69}$		$1*10^{-89}$		0.025
NW	Pear	6,303	reference	6,324	reference	6,280	reference	6,859	reference	6,485	reference
	Slim	5,562	-0.12 (-0.15 to -0.09)**	5,576	0.02 (-0.01 to 0.06)	5,540	0.05 (0.01 to 0.08)*	6,053	-0.03 (-0.07 to 0.00)*	5,711	0.99 (0.87 to 1.13)
	Wide	8,396	-0.16 (-0.19 to -0.13)**	8,431	-0.01 (-0.05 to 0.02)	8,360	-0.06 (-0.09 to -0.03)**	9,159	-0.15 (-0.18 to -0.12)**	8,589	0.97 (0.87 to 1.10)
	Apple	3,874	-0.27 (-0.31 to -0.24)**	3,890	0.00 (-0.04 to 0.04)	3,853	0.02 (-0.01 to 0.06)	4,196	-0.15 (-0.19 to -0.12)**	3,917	0.93 (0.80 to 1.08)
OW	Pear	10,119	-0.48 (-0.50 to -0.45)**	10,148	0.01 (-0.02 to 0.04)	10,079	-0.04 (-0.07 to -0.01)*	10,921	-0.32 (-0.35 to -0.29)**	10,240	1.04 (0.93 to 1.17)
	Slim	13,392	-0.58 (-0.61 to -0.56)**	13,434	0.06 (0.03 to 0.09)**	13,340	0.00 (-0.02 to 0.03)	14,502	-0.34 (-0.36 to -0.31)**	13,602	1.02 (0.91 to 1.13)
	Wide	16,588	-0.54 (-0.57 to -0.52)**	16,624	-0.01 (-0.04 to 0.02)	16,520	-0.12 (-0.15 to -0.09)**	17,949	-0.43 (-0.46 to -0.40)**	16,922	1.03 (0.93 to 1.15)
	Apple	11,371	-0.66 (-0.69 to -0.63)**	11,405	0.01 (-0.02 to 0.04)	11,323	-0.09 (-0.12 to -0.06)**	12,329	-0.46 (-0.49 to -0.44)**	11,588	0.93 (0.83 to 1.05)
OB	Pear	4,730	-0.77 (-0.80 to -0.74)**	4,759	-0.10 (-0.14 to -0.06)**	4,709	-0.32 (-0.35 to -0.28)**	5,125	-0.72 (-0.76 to -0.69)**	4,808	1.26 (1.10 to 1.43)*
	Slim	5,865	-0.86 (-0.89 to -0.82)**	5,907	-0.01 (-0.05 to 0.02)	5,847	-0.24 (-0.27 to -0.21)**	6,359	-0.70 (-0.73 to -0.66)**	6,002	1.17 (1.03 to 1.32)*
	Wide	9,259	-0.82 (-0.85 to -0.79)**	9,300	-0.19 (-0.22 to -0.15)**	9,203	-0.44 (-0.47 to -0.41)**	9,971	-0.85 (-0.88 to -0.82)**	9,394	1.29 (1.15 to 1.44)**
	Apple	6,078	-0.88 (-0.91 to -0.85)**	6,100	-0.10 (-0.14 to -0.07)**	6,056	-0.31 (-0.35 to -0.28)**	6,568	-0.78 (-0.81 to -0.75)**	6,180	1.18 (1.04 to 1.34)*
	p-value		$5*10^{-13}$		$1*10^{-7}$		$5*10^{-7}$		$2*10^{-7}$		0.831
**Women (overall)**											
ALL	Pear	45,202	reference	45,386	reference	45,050	reference	49,936	reference	46,958	reference
	Slim	40,334	-0.14 (-0.15 to -0.13)**	40,511	0.08 (0.07 to 0.09)**	40,170	0.02 (0.01 to 0.03)*	44,462	-0.02 (-0.03 to -0.01)*	41,770	0.99 (0.95 to 1.03)
	Wide	57,280	-0.25 (-0.26 to -0.24)**	57,471	0.00 (-0.01 to 0.01)	57,051	0.03 (0.01 to 0.04)**	63,100	-0.04 (-0.06 to -0.03)**	59,231	0.91 (0.87 to 0.94)**
	Apple	44,646	-0.44 (-0.45 to -0.43)**	44,834	0.10 (0.09 to 0.12)**	44,493	0.08 (0.06 to 0.09)**	49,113	-0.05 (-0.06 to -0.03)**	46,133	0.89 (0.85 to 0.92)**
	p-value		$<10^{-216}$		$4*10^{-94}$		$2*10^{-31}$		$3*10^{-16}$		$2*10^{-10}$
NW	Pear	21,998	reference	22,082	reference	21,927	reference	24,304	reference	22,851	reference
	Slim	18,244	-0.10 (-0.11 to -0.08)**	18,327	0.03 (0.01 to 0.05)*	18,171	0.02 (0.00 to 0.04)*	20,149	-0.01 (-0.03 to 0.01)	18,956	0.99 (0.94 to 1.05)
	Wide	21,271	-0.21 (-0.22 to -0.19)**	21,334	-0.01 (-0.03 to 0.01)	21,192	0.02 (0.00 to 0.04)*	23,551	-0.04 (-0.06 to -0.03)**	22,085	0.92 (0.86 to 0.97)*
	Apple	13,925	-0.39 (-0.40 to -0.37)**	13,988	0.02 (0.00 to 0.04)	13,871	0.08 (0.06 to 0.10)**	15,391	-0.04 (-0.06 to -0.02)*	14,506	0.92 (0.86 to 0.98)*
OW	Pear	14,994	-0.38 (-0.40 to -0.36)**	15,063	-0.22 (-0.24 to -0.20)**	14,946	0.25 (0.23 to 0.27)**	16,617	0.13 (0.11 to 0.15)**	15,633	1.03 (0.97 to 1.10)
	Slim	15,065	-0.57 (-0.59 to -0.55)**	15,128	-0.11 (-0.13 to -0.09)**	15,004	0.29 (0.27 to 0.31)**	16,631	0.12 (0.10 to 0.13)**	15,588	1.03 (0.97 to 1.10)
	Wide	20,216	-0.69 (-0.71 to -0.67)**	20,280	-0.20 (-0.22 to -0.18)**	20,135	0.30 (0.28 to 0.32)**	22,203	0.10 (0.08 to 0.12)**	20,886	0.91 (0.85 to 0.97)*
	Apple	19,514	-0.91 (-0.93 to -0.89)**	19,588	-0.08 (-0.10 to -0.06)**	19,458	0.35 (0.33 to 0.37)**	21,482	0.09 (0.07 to 0.10)**	20,144	0.91 (0.86 to 0.97)*
OB	Pear	8,210	-0.95 (-0.97 to -0.93)**	8,241	-0.50 (-0.53 to -0.48)**	8,177	0.54 (0.52 to 0.57)**	9,015	0.27 (0.24 to 0.29)**	8,474	1.14 (1.05 to 1.23)*
	Slim	7,025	-1.11 (-1.14 to -1.09)**	7,056	-0.35 (-0.37 to -0.32)**	6,995	0.53 (0.51 to 0.56)**	7,682	0.21 (0.19 to 0.24)**	7,226	1.10 (1.01 to 1.20)*
	Wide	15,793	-1.20 (-1.22 to -1.18)**	15,857	-0.50 (-0.52 to -0.48)**	15,724	0.54 (0.52 to 0.56)**	17,346	0.20 (0.18 to 0.22)**	16,260	1.05 (0.97 to 1.12)
	Apple	11,207	-1.35 (-1.37 to -1.33)**	11,258	-0.31 (-0.33 to -0.29)**	11,164	0.58 (0.55 to 0.60)**	12,240	0.20 (0.18 to 0.23)**	11,483	0.95 (0.88 to 1.02)
	p-value		$1*10^{-32}$		$8*10^{-36}$		0.006		0.178		0.426

Group		SHBG Count	SHBG SD_{diff} (95% CI)	Albumin Count	Albumin SD_{diff} (95% CI)	Testosterone Count	Testosterone SD_{diff} (95% CI)	Free Testosterone Count	Free Testosterone SD_{diff} (95% CI)	Oestradiol (binary) Count	Oestradiol (binary) OR (95% CI)
**Women Pre-MP**											
ALL	Pear	11,014	reference	11,039	reference	10,978	reference	12,196	reference	11,483	reference
	Slim	10,162	-0.10 (-0.12 to -0.07)**	10,197	0.07 (0.04 to 0.10)**	10,124	0.02 (0.00 to 0.04)	11,290	-0.01 (-0.03 to 0.01)	10,615	0.98 (0.91 to 1.04)
	Wide	8,491	-0.19 (-0.21 to -0.17)**	8,518	0.00 (-0.03 to 0.03)	8,463	0.02 (0.00 to 0.05)*	9,409	-0.03 (-0.05 to -0.01)*	8,790	0.92 (0.86 to 0.99)*
	Apple	7,181	-0.36 (-0.39 to -0.34)**	7,204	0.08 (0.05 to 0.11)**	7,155	0.09 (0.06 to 0.11)**	7,929	-0.01 (-0.03 to 0.01)	7,436	0.88 (0.82 to 0.95)*
	p-value		$7*10^{-197}$		$8*10^{-12}$		$2*10^{-12}$		0.036		0.003
NW	Pear	6,076	reference	6,086	reference	6,055	reference	6,742	reference	6,370	reference
	Slim	5,224	-0.06 (-0.09 to -0.03)*	5,243	0.03 (0.00 to 0.07)	5,201	0.02 (-0.01 to 0.04)	5,807	0.00 (-0.03 to 0.03)	5,463	0.94 (0.86 to 1.04)
	Wide	4,160	-0.15 (-0.18 to -0.12)**	4,173	0.02 (-0.02 to 0.05)	4,152	0.01 (-0.02 to 0.04)	4,626	-0.03 (-0.06 to 0.00)*	4,319	0.95 (0.86 to 1.05)
	Apple	2,702	-0.29 (-0.33 to -0.25)**	2,712	-0.01 (-0.05 to 0.04)	2,688	0.07 (0.03 to 0.10)*	2,994	-0.01 (-0.05 to 0.02)	2,808	0.89 (0.80 to 1.00)*
OW	Pear	3,241	-0.36 (-0.39 to -0.32)**	3,253	-0.25 (-0.29 to -0.21)**	3,233	0.22 (0.19 to 0.26)**	3,586	0.11 (0.08 to 0.14)**	3,369	0.96 (0.87 to 1.07)
	Slim	3,357	-0.51 (-0.55 to -0.48)**	3,368	-0.14 (-0.18 to -0.10)**	3,344	0.27 (0.23 to 0.30)**	3,728	0.11 (0.08 to 0.14)**	3,515	0.99 (0.89 to 1.10)
	Wide	2,474	-0.62 (-0.66 to -0.58)**	2,483	-0.27 (-0.31 to -0.22)**	2,460	0.27 (0.24 to 0.31)**	2,729	0.09 (0.05 to 0.12)**	2,563	0.90 (0.80 to 1.01)
	Apple	2,839	-0.82 (-0.85 to -0.78)**	2,847	-0.14 (-0.19 to -0.10)**	2,833	0.36 (0.33 to 0.39)**	3,137	0.12 (0.09 to 0.15)**	2,929	0.86 (0.77 to 0.96)*
OB	Pear	1,697	-0.98 (-1.02 to -0.93)**	1,700	-0.61 (-0.66 to -0.56)**	1,690	0.49 (0.45 to 0.53)**	1,868	0.20 (0.16 to 0.24)**	1,744	0.91 (0.79 to 1.04)
	Slim	1,581	-1.09 (-1.14 to -1.05)**	1,586	-0.49 (-0.55 to -0.44)**	1,579	0.46 (0.42 to 0.51)**	1,755	0.14 (0.10 to 0.18)**	1,637	0.88 (0.77 to 1.01)
	Wide	1,857	-1.18 (-1.22 to -1.14)**	1,862	-0.61 (-0.66 to -0.56)**	1,851	0.49 (0.45 to 0.53)**	2,054	0.15 (0.11 to 0.19)**	1,908	0.76 (0.67 to 0.86)**
	Apple	1,640	-1.35 (-1.40 to -1.31)**	1,645	-0.40 (-0.45 to -0.35)**	1,634	0.53 (0.49 to 0.57)**	1,798	0.15 (0.10 to 0.19)**	1,699	0.76 (0.66 to 0.87)**
	p-value		$3*10^{-7}$		$7*10^{-9}$		0.059		0.256		0.628

Group		Free Oestradiol Count	Free Oestradiol SD_{diff} (95% CI)	Oestradiol (imputed) Count	Oestradiol (imputed) SD_{diff} (95% CI)
**Women Pre-MP**					
ALL	Pear	10,277	reference	11,483	reference
	Slim	9,470	0.00 (-0.02 to 0.03)	10,615	-0.01 (-0.04 to 0.01)
	Wide	7,853	-0.01 (-0.04 to 0.02)	8,790	-0.04 (-0.07 to -0.01)*
	Apple	6,680	0.00 (-0.03 to 0.03)	7,436	-0.07 (-0.10 to -0.04)**
	p-value		0.808		$8*10^{-6}$
NW	Pear	5,692	reference	6,370	reference
	Slim	4,865	-0.02 (-0.05 to 0.02)	5,463	-0.03 (-0.06 to 0.01)
	Wide	3,847	0.00 (-0.04 to 0.04)	4,319	-0.02 (-0.06 to 0.01)
	Apple	2,512	-0.01 (-0.06 to 0.03)	2,808	-0.07 (-0.11 to -0.03)*
OW	Pear	3,019	0.06 (0.01 to 0.10)*	3,369	-0.04 (-0.08 to 0.00)
	Slim	3,138	0.10 (0.05 to 0.14)**	3,515	-0.02 (-0.06 to 0.02)
	Wide	2,300	0.04 (0.00 to 0.09)	2,563	-0.09 (-0.14 to -0.05)**
	Apple	2,630	0.06 (0.02 to 0.11)*	2,929	-0.11 (-0.15 to -0.06)**
OB	Pear	1,566	0.11 (0.06 to 0.17)**	1,744	-0.11 (-0.16 to -0.05)**
	Slim	1,467	0.11 (0.06 to 0.17)**	1,637	-0.12 (-0.17 to -0.06)**
	Wide	1,706	0.08 (0.03 to 0.13)*	1,908	-0.17 (-0.22 to -0.12)**
	Apple	1,538	0.12 (0.07 to 0.18)**	1,699	-0.16 (-0.22 to -0.11)**
	p-value		0.365		0.349

**Group**		**SHBG**		**Albumin**		**Testosterone**		**Free Testosterone**		**Oestradiol** (binary)	
		Count	SD_{diff} (95% CI)	Count	SD_{diff} (95% CI)	Count	SD_{diff} (95% CI)	Count	SD_{diff} (95% CI)	Count	OR (95% CI)
**Women Post-MP Never-HRT**											
ALL	Pear	12,878	reference	12,938	reference	12,842	reference	14,177	reference	13,351	reference
	Slim	11,274	-0.15 (-0.17 to -0.13)**	11,325	0.09 (0.07 to 0.12)**	11,223	0.02 (0.00 to 0.05)	12,372	-0.02 (-0.04 to 0.00)	11,623	0.95 (0.83 to 1.09)
	Wide	18,893	-0.27 (-0.29 to -0.26)**	18,942	0.00 (-0.02 to 0.02)	18,821	0.02 (0.00 to 0.04)*	20,772	-0.05 (-0.07 to -0.03)**	19,489	0.98 (0.87 to 1.11)
	Apple	14,140	-0.49 (-0.51 to -0.47)**	14,199	0.11 (0.08 to 0.13)**	14,090	0.07 (0.05 to 0.09)**	15,543	-0.06 (-0.09 to -0.04)**	14,624	0.88 (0.77 to 1.01)
	p-value		$<10^{-216}$		$4*10^{-35}$		$2*10^{-8}$		$1*10^{-8}$		0.263
NW	Pear	6,227	reference	6,254	reference	6,209	reference	6,852	reference	6,447	reference
	Slim	5,120	-0.09 (-0.12 to -0.06)**	5,137	0.04 (0.01 to 0.08)*	5,100	0.04 (0.01 to 0.07)*	5,655	0.01 (-0.02 to 0.05)	5,305	1.01 (0.82 to 1.23)
	Wide	6,857	-0.23 (-0.26 to -0.20)**	6,864	0.01 (-0.02 to 0.04)	6,823	0.03 (0.00 to 0.07)*	7,586	-0.03 (-0.06 to 0.00)*	7,116	1.04 (0.86 to 1.26)
	Apple	4,347	-0.42 (-0.46 to -0.39)**	4,374	0.03 (-0.01 to 0.07)	4,332	0.09 (0.06 to 0.13)**	4,795	-0.03 (-0.07 to 0.00)	4,529	0.83 (0.66 to 1.06)
OW	Pear	4,276	-0.40 (-0.43 to -0.37)**	4,301	-0.20 (-0.24 to -0.16)**	4,264	0.29 (0.25 to 0.32)**	4,729	0.18 (0.14 to 0.21)**	4,454	1.15 (0.93 to 1.42)
	Slim	4,250	-0.62 (-0.65 to -0.59)**	4,269	-0.08 (-0.12 to -0.04)**	4,230	0.30 (0.26 to 0.34)**	4,649	0.12 (0.09 to 0.16)**	4,365	1.17 (0.95 to 1.45)
	Wide	6,790	-0.72 (-0.75 to -0.69)**	6,811	-0.18 (-0.21 to -0.15)**	6,768	0.31 (0.28 to 0.35)**	7,467	0.12 (0.09 to 0.15)**	7,016	1.08 (0.88 to 1.32)
	Apple	6,259	-0.98 (-1.01 to -0.95)**	6,279	-0.05 (-0.08 to -0.02)*	6,241	0.35 (0.32 to 0.39)**	6,885	0.08 (0.05 to 0.11)**	6,449	1.09 (0.89 to 1.34)
OB	Pear	2,375	-0.93 (-0.97 to -0.89)**	2,383	-0.47 (-0.51 to -0.42)**	2,369	0.58 (0.53 to 0.62)**	2,596	0.31 (0.27 to 0.36)**	2,450	1.82 (1.45 to 2.30)**
	Slim	1,904	-1.12 (-1.16 to -1.08)**	1,919	-0.30 (-0.34 to -0.25)**	1,893	0.57 (0.52 to 0.62)**	2,068	0.26 (0.21 to 0.31)**	1,953	1.40 (1.08 to 1.83)*
	Wide	5,246	-1.24 (-1.27 to -1.21)**	5,267	-0.49 (-0.53 to -0.46)**	5,230	0.56 (0.53 to 0.60)**	5,719	0.23 (0.19 to 0.26)**	5,357	1.72 (1.41 to 2.09)**
	Apple	3,534	-1.40 (-1.43 to -1.36)**	3,546	-0.31 (-0.35 to -0.27)**	3,517	0.61 (0.57 to 0.65)**	3,863	0.23 (0.19 to 0.27)**	3,646	1.49 (1.19 to 1.86)*
	p-value		$5*10^{-12}$		$7*10^{-11}$		0.507		0.050		0.493
**Women Post-MP Past-HRT**											
ALL	Pear	10,346	reference	10,382	reference	10,308	reference	11,390	reference	10,674	reference
	Slim	8,982	-0.15 (-0.17 to -0.12)**	9,014	0.08 (0.05 to 0.11)**	8,944	0.02 (-0.01 to 0.05)	9,849	-0.02 (-0.05 to 0.00)	9,267	1.15 (0.93 to 1.40)
	Wide	17,147	-0.28 (-0.30 to -0.26)**	17,200	-0.01 (-0.03 to 0.02)	17,074	0.02 (-0.01 to 0.04)	18,894	-0.06 (-0.09 to -0.04)**	17,808	1.04 (0.86 to 1.24)
	Apple	12,972	-0.45 (-0.47 to -0.43)**	13,029	0.11 (0.08 to 0.13)**	12,927	0.08 (0.05 to 0.10)**	14,225	-0.05 (-0.08 to -0.02)*	13,354	1.02 (0.84 to 1.23)
	p-value		$<10^{-216}$		$2*10^{-29}$		$4*10^{-8}$		$6*10^{-6}$		0.560
NW	Pear	4,447	reference	4,464	reference	4,435	reference	4,890	reference	4,560	reference
	Slim	3,563	-0.11 (-0.14 to -0.07)**	3,576	0.02 (-0.03 to 0.06)	3,551	-0.01 (-0.05 to 0.04)	3,905	-0.04 (-0.08 to 0.01)	3,690	1.35 (0.93 to 1.98)
	Wide	5,663	-0.23 (-0.27 to -0.20)**	5,679	0.00 (-0.04 to 0.04)	5,638	-0.01 (-0.05 to 0.03)	6,256	-0.08 (-0.12 to -0.04)**	5,899	1.26 (0.88 to 1.79)
	Apple	3,737	-0.41 (-0.45 to -0.38)**	3,746	0.03 (-0.01 to 0.07)	3,724	0.06 (0.02 to 0.11)*	4,123	-0.07 (-0.11 to -0.02)*	3,884	1.50 (1.03 to 2.17)*
OW	Pear	3,843	-0.39 (-0.42 to -0.35)**	3,855	-0.17 (-0.22 to -0.13)**	3,826	0.23 (0.18 to 0.27)**	4,257	0.12 (0.07 to 0.16)**	4,001	1.25 (0.86 to 1.83)
	Slim	3,733	-0.56 (-0.59 to -0.52)**	3,747	-0.07 (-0.11 to -0.03)*	3,718	0.30 (0.25 to 0.34)**	4,114	0.13 (0.09 to 0.18)**	3,845	1.83 (1.28 to 2.61)*
	Wide	6,538	-0.71 (-0.75 to -0.68)**	6,557	-0.18 (-0.21 to -0.14)**	6,511	0.29 (0.25 to 0.33)**	7,170	0.09 (0.05 to 0.13)**	6,768	1.52 (1.09 to 2.13)*
	Apple	5,995	-0.91 (-0.94 to -0.88)**	6,023	-0.06 (-0.10 to -0.03)*	5,975	0.34 (0.30 to 0.38)**	6,569	0.09 (0.05 to 0.13)**	6,168	1.62 (1.16 to 2.27)*
OB	Pear	2,056	-0.92 (-0.96 to -0.88)**	2,063	-0.44 (-0.49 to -0.39)**	2,047	0.55 (0.49 to 0.60)**	2,243	0.28 (0.23 to 0.33)**	2,113	3.34 (2.33 to 4.78)**
	Slim	1,686	-1.12 (-1.16 to -1.07)**	1,691	-0.28 (-0.33 to -0.22)**	1,675	0.51 (0.45 to 0.57)**	1,830	0.20 (0.15 to 0.26)**	1,732	2.60 (1.76 to 3.85)**
	Wide	4,946	-1.18 (-1.22 to -1.15)**	4,964	-0.44 (-0.48 to -0.40)**	4,925	0.53 (0.49 to 0.57)**	5,468	0.20 (0.16 to 0.24)**	5,141	2.55 (1.84 to 3.53)**
	Apple	3,240	-1.31 (-1.35 to -1.28)**	3,260	-0.23 (-0.27 to -0.18)**	3,228	0.58 (0.53 to 0.63)**	3,533	0.23 (0.18 to 0.28)**	3,302	2.01 (1.40 to 2.88)*
	p-value		$2*10^{-9}$		$3*10^{-10}$		0.071		0.248		0.006

Group		SHBG Count	SHBG SD$_{diff}$ (95% CI)	Albumin Count	Albumin SD$_{diff}$ (95% CI)	Testosterone Count	Testosterone SD$_{diff}$ (95% CI)	Free Testosterone Count	Free Testosterone SD$_{diff}$ (95% CI)	Oestradiol (binary) Count	Oestradiol (binary) OR (95% CI)
**Women Post-MP Current-HRT**											
ALL	Pear	2,323	reference	2,344	reference	2,311	reference	2,593	reference	2,457	reference
	Slim	2,075	-0.17 (-0.24 to -0.10)**	2,095	0.03 (-0.03 to 0.09)	2,071	0.01 (-0.05 to 0.08)	2,294	-0.05 (-0.11 to 0.01)	2,154	1.01 (0.89 to 1.15)
	Wide	2,898	-0.25 (-0.32 to -0.19)**	2,916	-0.01 (-0.07 to 0.04)	2,886	0.03 (-0.04 to 0.09)	3,197	-0.05 (-0.11 to 0.01)	2,986	0.94 (0.83 to 1.05)
	Apple	2,359	-0.49 (-0.56 to -0.42)**	2,376	0.12 (0.06 to 0.18)**	2,354	0.08 (0.01 to 0.14)*	2,597	-0.07 (-0.13 to 0.00)*	2,430	0.86 (0.76 to 0.98)*
	p-value		$3*10^{-43}$		$6*10^{-6}$		0.122		0.164		0.054
NW	Pear	1,211	reference	1,223	reference	1,203	reference	1,354	reference	1,281	reference
	Slim	1,020	-0.13 (-0.23 to -0.03)*	1,031	0.02 (-0.07 to 0.10)	1,019	0.05 (-0.04 to 0.15)	1,113	0.01 (-0.08 to 0.10)	1,048	0.96 (0.80 to 1.14)
	Wide	1,203	-0.23 (-0.33 to -0.14)**	1,212	-0.01 (-0.09 to 0.08)	1,201	0.01 (-0.08 to 0.10)	1,333	-0.06 (-0.14 to 0.03)	1,238	0.88 (0.74 to 1.04)
	Apple	869	-0.43 (-0.54 to -0.33)**	874	0.07 (-0.02 to 0.16)	869	0.05 (-0.05 to 0.15)	950	-0.09 (-0.18 to 0.00)	891	0.83 (0.68 to 1.00)*
OW	Pear	779	-0.31 (-0.42 to -0.20)**	784	-0.17 (-0.26 to -0.08)*	776	0.23 (0.13 to 0.33)**	871	0.12 (0.03 to 0.22)*	824	0.93 (0.77 to 1.13)
	Slim	771	-0.59 (-0.69 to -0.48)**	778	-0.16 (-0.25 to -0.07)*	769	0.21 (0.11 to 0.32)**	873	0.02 (-0.07 to 0.12)	811	0.97 (0.80 to 1.18)
	Wide	1,062	-0.65 (-0.74 to -0.55)**	1,065	-0.15 (-0.24 to -0.07)*	1,057	0.30 (0.21 to 0.40)**	1,170	0.10 (0.01 to 0.19)*	1,096	0.90 (0.75 to 1.07)
	Apple	1,038	-0.86 (-0.96 to -0.76)**	1,050	-0.02 (-0.10 to 0.07)	1,035	0.31 (0.21 to 0.40)**	1,162	0.05 (-0.04 to 0.14)	1,078	0.83 (0.69 to 1.00)*
OB	Pear	333	-0.79 (-0.93 to -0.64)**	337	-0.35 (-0.48 to -0.23)**	332	0.44 (0.30 to 0.58)**	368	0.20 (0.07 to 0.33)*	352	0.86 (0.67 to 1.12)
	Slim	284	-0.87 (-1.03 to -0.72)**	286	-0.22 (-0.36 to -0.09)*	283	0.39 (0.24 to 0.54)**	308	0.08 (-0.06 to 0.22)	295	1.02 (0.78 to 1.34)
	Wide	633	-0.97 (-1.08 to -0.85)**	639	-0.42 (-0.52 to -0.32)**	628	0.41 (0.30 to 0.52)**	694	0.12 (0.01 to 0.22)*	652	0.92 (0.74 to 1.13)
	Apple	452	-1.31 (-1.44 to -1.18)**	452	-0.17 (-0.29 to -0.06)*	450	0.55 (0.43 to 0.68)**	485	0.18 (0.06 to 0.30)*	461	0.81 (0.64 to 1.03)
	p-value		0.249		0.163		0.371		0.243		0.93

ABSI – a body shape index (cut-offs ≥80 for men, ≥73 for women); **Apple** – large-ABSI-small-HI; **BMI** – body mass index; **Count** – number of participants with available biomarker measurements per category; **HI** – hip index (cut-offs ≥49 for men, ≥64 for women); **HRT** – hormone replacement therapy; **NW** – normal weight (BMI≥18.5 to <25 kg/m^2); **OB** – obese (BMI≥30 to <45 kg/m^2); **OR** – odds ratio; **OW** – overweight (BMI≥25 to <30 kg/m^2); **Pear** – small-ABSI-large-HI; **Post-MP** – post-menopausal; **Pre-MP** – pre-menopausal; **SHBG** – sex hormone binding globulin; **Slim** – small-ABSI-small-HI; **Wide** – large-ABSI-large-HI.

SD$_{diff}$ (95% CI) – estimates for standard deviation differences (95% confidence interval) were obtained from multivariable linear regression models including SHBG, albumin, total testosterone, free testosterone, or total or free oestradiol (Pre-MP) on a continuous standard deviation scale (sex-specific z-scores) as an outcome variable and as independent variables ABSI-by-HI cross-classification and BMI categories (ALL, reference "pear"), or an BMI-by-ABSI-by-HI cross-classification variable (NW, OW, and OB, reference "pear"-NW); **OR (95% CI)** – estimates for odds ratios of oestradiol detection (95% confidence interval) were obtained from multivariable logistic regression models. All models were adjusted for height, age at enrolment, weight change within the last year preceding enrolment, smoking status, alcohol consumption, physical activity, Townsend deprivation

index, region of the assessment centre, time of blood collection, fasting time, use of cholesterol lowering drugs (except Pre-MP), and in women also age at the last live birth, oral contraceptives use with time since stopped, bilateral oophorectomy (except Pre-MP) and, additionally, menopausal status and HRT use and duration (women overall), time of the menstrual period (Pre-MP), time since stopped and duration of HRT use (Past-HRT), or duration of use and type of HRT (Current-HRT). Participant groups are defined in Supplementary Figure S1. Covariates are defined in Supplementary Methods.

p-value (ALL) – obtained from a likelihood ratio test comparing a model including only a categorical variable for BMI and covariates with a model additionally including an ABSI-by-HI cross-classification variable (evaluates body shape overall).

p-value (NW, OW, OB) – obtained from a likelihood ratio test comparing an additive model including an ABSI-by-HI cross-classification variable, BMI categories, and covariates with the interaction model including the BMI-by-ABSI-by-HI cross-classification variable (evaluates heterogeneity by BMI).

* – $p<0.05$ from Wald test for the individual term; ** – $p<0.0001$.

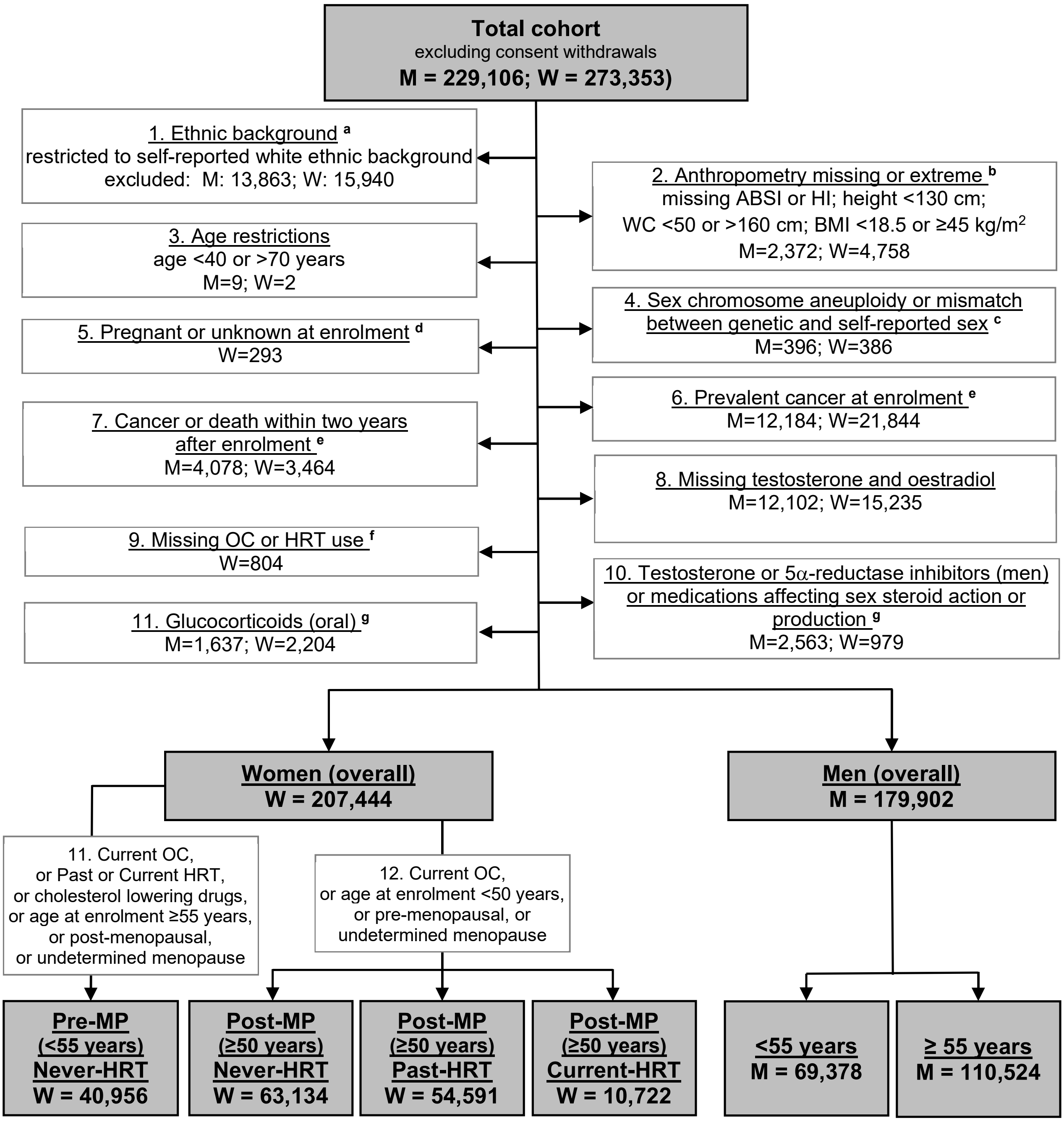

Supplementary Figure S1 Flow diagram of UK Biobank participants included in the study

ABSI – a body shape index; **BMI** – body mass index; **HI** – hip index; **HRT** – hormone replacement therapy; **M** – number of men; **OC** – oral contraceptives; **W** – number of women; **WC** – waist circumference. Supplementary Methods include details of the definition of variables. The exclusion criteria were applied sequentially in the displayed order, such that each excluded individual was counted only once. Specific fields used to define the exclusions are listed below:

[a] – Field [21000-0.0] "*Ethnic background*"; retained in the study were codes: 1 "*White*", 1001 "*British*", 1002 "*Irish*", 1003 "*Any other white background*".

[b] – anthropometric measurements were obtained from Fields [48-0/2.0] "*Waist circumference*", Field [49-0/2.0] "*Hip circumference*", Field [50-0/2.0] "*Standing height*" and Field [21002-0/2.0] "*Weight*".

[c] – excluded were participants with code 1 for Field [22019-0.0] "*Sex chromosome aneuploidy*" OR with a mismatch between Field [22001-0.0] "*Genetic sex*" and Field [34-0.0] "*Sex (self-reported)*".

[d] – Field [3140-0.0] "*Pregnant*"; Answer: 1 "Yes", OR Answer 2: "Unsure", OR Missing.

[e] – prevalent and incident cancer cases and deaths were ascertained according to our previous publication [ref. 3].

[f] – the definitions of oral contraceptives and HRT use are described in Supplementary Methods.

[g] – Supplementary Table S1 includes a list of medications for exclusion.

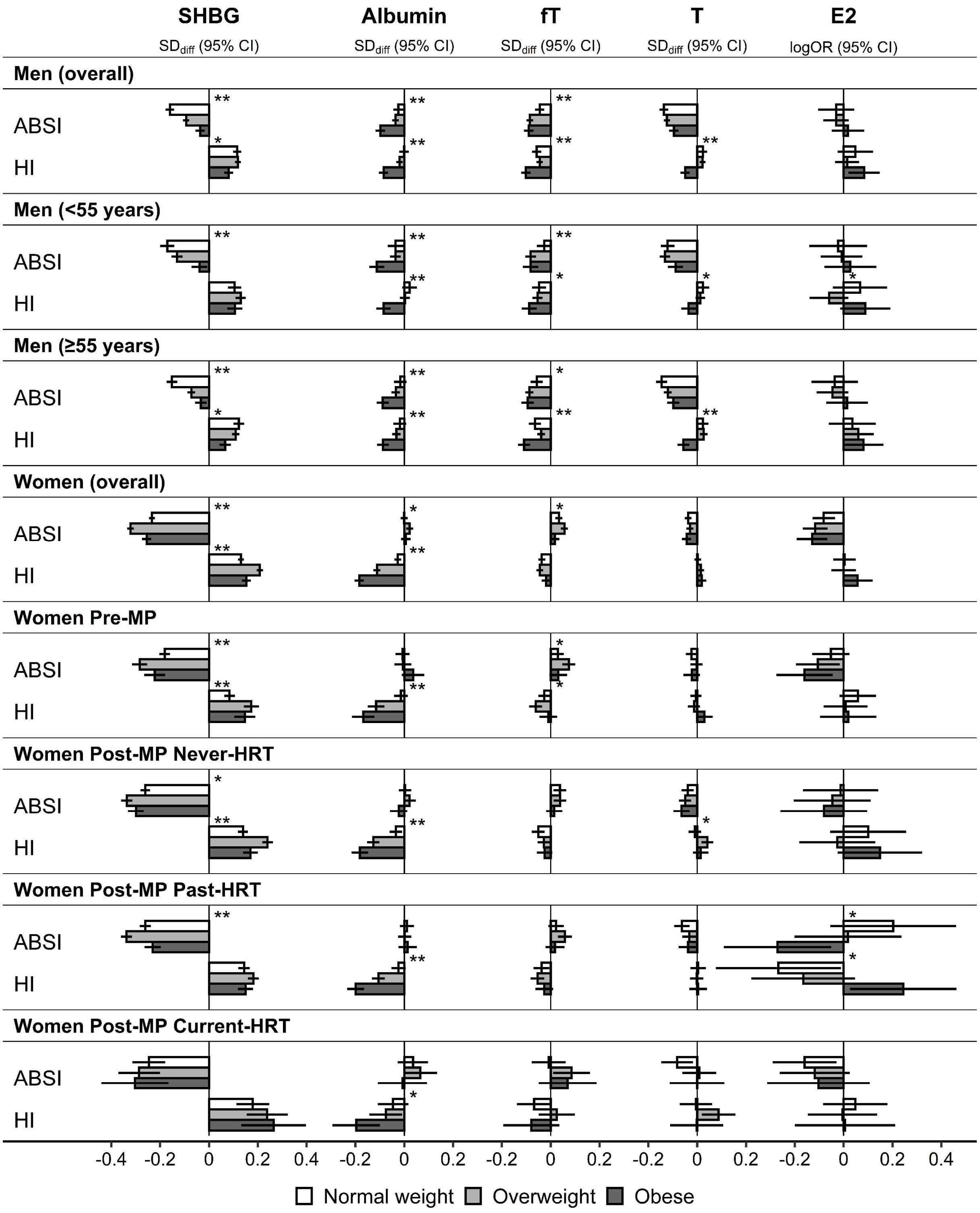

Supplementary Figure S2 Associations of body shape indices with sex steroids and their binding proteins: heterogeneity by BMI

ABSI – a body shape index (large vs small, cut-offs ≥80 for men, ≥73 for women); **BMI** – body mass index; **E2** – oestradiol; **fT** – free testosterone; **HI** – hip index (large vs small, cut-offs ≥49 for men, ≥64 for women); **HRT** – hormone replacement therapy; **Normal weight** – BMI≥18.5 to BMI<25 kg/m^2; **Obese** – BMI≥30 to BMI<45 kg/m^2; **OR** – odds ratio; **Overweight** – BMI≥25 to

BMI<30 kg/m^2; **Post-MP** – post-menopausal; **Pre-MP** – pre-menopausal; **SHBG** – sex hormone binding globulin; **T** – total testosterone.

SD_{diff} (95% CI) – estimates for standard deviation differences (95% confidence interval) were obtained from multivariable linear regression models including SHBG, albumin, T, or fT (continuous standard deviation scale, sex-specific z-scores) as an outcome variable; **logOR (95% CI)** – estimates for odds ratios of oestradiol detection (95% confidence interval) were obtained from multivariable logistic regression models (logOR corresponds to the linear predictor of the model). All models in included dichotomised ABSI and HI (large vs small) as independent variables and were adjusted for BMI categories, height, age at enrolment, weight change within the last year preceding enrolment, smoking status, alcohol consumption, physical activity, Townsend deprivation index, region of the assessment centre, time of blood collection, fasting time, use of cholesterol lowering drugs (except Pre-MP), and in women age at the last live birth, oral contraceptives use with time since stopped, bilateral oophorectomy (except Pre-MP) and, additionally, menopausal status and HRT use and duration (women overall), time of the menstrual period (Pre-MP), time since stopped and duration of HRT use (Past-HRT), or duration of use and type of HRT (Current-HRT). Participant groups are defined in Supplementary Figure S1. Covariates are defined in Supplementary Methods.

* – p<0.05 from a likelihood ratio test comparing the additive model including dichotomised ABHI and HI, BMI categories, and covariates with an interaction model including an interaction term between ABSI and BMI (or between HI and BMI) and covariates (evaluates heterogeneity by BMI); ** – p<0.0001.

References

The numbers of the cited references correspond to the main document.

3. Christakoudi, S., Tsilidis, K. K., Evangelou, E., Riboli, E. A Body Shape Index (ABSI), hip index and risk of cancer in the UK Biobank cohort. *Cancer Med.* **10,** 5614-5628; 10.1002/CAM4.4097 (2021).

6. Tin Tin, S., Reeves, G. K. & Key, T. J. Body size and composition, physical activity and sedentary time in relation to endogenous hormones in premenopausal and postmenopausal women: Findings from the UK Biobank. *Int. J. Cancer.* **147**, 2101-2115 (2020).

11. Christakoudi, S., Tsilidis, K. K., Evangelou, E. & Riboli, E. Association of body-shape phenotypes with imaging measures of body composition in the UK Biobank cohort: relevance to colon cancer risk. *BMC Cancer* **21,** 1106; 10.1186/s12885-021-08820-6 (2021).

16. Sodergard, R., Backstrom, T., Shanbhag, V. & Carstensen, H. Calculation of free and bound fractions of testosterone and estradiol-17 beta to human plasma proteins at body temperature. *J. Steroid. Biochem.* **16**, 801-810 (1982).
